# Microenvironment‐Responsive Biomimetic Hydrogels Delivering Cistanche Deserticola‐Derived Nanovesicles for Intervertebral Disc Degeneration

**DOI:** 10.1002/advs.78698

**Published:** 2026-11-09

**Authors:** Ning Xu, Xiaoyu Cai, Xiaofu Tang, Weidong Liang, Linsong Teng, Yudong Jing, Haifeng Hu, Yuhao Duan, Mingjie Kuang, Lei Zhang, Shijie Han, Weibin Sheng

**Affiliations:** ^1^ Department of Spine Surgery The First Affiliated Hospital of Xinjiang Medical University Urumqi People's Republic of China; ^2^ Department of Orthopedics Provincial Hospital Affiliated to Shandong First Medical University Jinan People's Republic of China; ^3^ Second Clinical Medical College of Shandong University of Traditional Chinese Medicine Jinan People's Republic of China; ^4^ Department of Nuclear Medicine The First Affiliated Hospital of Shandong First Medical University & Shandong Provincial Qianfoshan Hospital Jinan People's Republic of China; ^5^ Department of Orthopedic Surgery Tissue Engineering Laboratory Department of Radiology The First Affiliated Hospital of Shandong First Medical University & Shandong Provincial Qianfoshan Hospital Shandong First Medical University Jinan People's Republic of China

**Keywords:** biomimetic scaffold, cistanche deserticola‐derived nanovesicles, intervertebral disc degeneration, nucleus pulposus cell senescence, pH/ROS dual‐responsive hydrogel

## Abstract

Intervertebral disc degeneration (IVDD) is driven by persistent oxidative stress and tissue acidosis, accelerating nucleus pulposus cell (NPC) senescence and extracellular matrix (ECM) degradation. While plant‐derived nanovesicles (PDNVs) hold therapeutic promise, rapid clearance and mechanical leakage constrain their translation. Here, natural nanovesicles from *Cistanche deserticola* (CDNVs) were incorporated into an injectable, microenvironment‐responsive hydrogel (HAPOCS@CDNVs) for localized, sustained delivery. Dynamic Schiff base and phenylboronate ester crosslinks provide biomechanical buffering, scavenge reactive oxygen species (ROS), and trigger on‐demand CDNV release under acidic and oxidative conditions. Mechanistically, CDNVs alleviate oxidative stress, support mitochondrial function, and inhibit NF‐κB activation, thereby suppressing the senescence‐associated secretory phenotype (SASP) and preserving ECM homeostasis. In a rat IVDD model, a single intradiscal injection of HAPOCS@CDNVs sustained disc height, preserved tissue hydration, and retarded degeneration over 8 weeks. This platform provides a promising, minimally invasive strategy for disc repair.

## Graphical abstract

An injectable, microenvironment‐responsive hydrogel (HAPOCS@CDNVs) is developed for sustained delivery of *Cistanche deserticola*‐derived nanovesicles (CDNVs). By scavenging ROS, restoring mitochondrial function, and attenuating SASP, this platform effectively suppresses nucleus pulposus cell senescence and facilitates extracellular matrix reconstruction, offering a minimally invasive strategy for intervertebral disc degeneration.

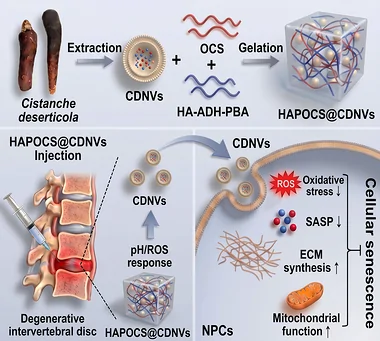

## 1 Introduction

Intervertebral disc degeneration (IVDD) represents a primary etiology of chronic low back pain, imposing a substantial socioeconomic burden and markedly diminishing patients' quality of life [1, 2]. The intervertebral disc is a complex fibrocartilaginous structure comprising the central nucleus pulposus (NP), the surrounding annulus fibrosus (AF), and the cartilaginous endplates (CEP) [3, 4]. Within the central region, nucleus pulposus cells (NPCs) are essential for synthesizing and maintaining the extracellular matrix (ECM), which sustains the hydration, biomechanical resilience, and tissue homeostasis of the disc [5]. During IVDD progression, abnormal mechanical loading and nutritional deficiency perturb the local microenvironment, triggering excessive accumulation of reactive oxygen species (ROS) and localized acidosis, thereby disrupting cellular redox equilibrium [6, 7]. Substantial evidence indicates that oxidative stress acts as a central driver of NPC damage by promoting lipid peroxidation, impairing mitochondrial function, and accelerating premature cellular senescence [8, 9]. Senescent NPCs progressively acquire a senescence‐associated secretory phenotype (SASP), characterized by the robust release of pro‐inflammatory cytokines and matrix‐degrading enzymes that accelerate the catabolic breakdown of core ECM components, particularly type II collagen [10]. Consequently, mitigating oxidative stress to preserve mitochondrial function and alleviate premature NPC senescence represents a promising therapeutic strategy for retarding disc degeneration.

Natural products serve as an abundant repository of bioactive compounds with well‐documented antioxidant and anti‐aging properties [11, 12]. Recently, plant‐derived nanovesicles (PDNVs) have emerged as promising natural nanotherapeutics and delivery platforms in regenerative medicine [13]. Compared with conventional mammalian extracellular vesicles, PDNVs offer scalable yield, favorable biocompatibility, and minimal immunogenicity [14, 15]. Furthermore, PDNVs inherently encapsulate active phytochemicals and can be efficiently internalized by mammalian cells to remodel cellular redox homeostasis [16]. For instance, nanovesicles isolated from plants such as ginger, grapefruit, and ginseng have demonstrated remarkable anti‐inflammatory, radical‐scavenging, and pro‐regenerative capacities across diverse disease models [17, 18, 19]. *Cistanche deserticola*, a traditional tonic herb long recognized for its robust antioxidative and anti‐senescent bioactivities [20, 21], represents an attractive source for therapeutic nanovesicles. However, utilizing *Cistanche deserticola*‐derived nanovesicles (CDNVs) to alleviate IVDD has yet to be explored. Despite their therapeutic promise, direct clinical translation of free CDNVs faces substantial local delivery hurdles. Because the intervertebral disc is the largest avascular structure in the human body and continuously subjected to dynamic mechanical loading, unencapsulated CDNVs directly injected into the disc space are prone to rapid leakage along the injection tract and premature displacement [22]. Moreover, free CDNVs exhibit a short biological half‐life and are vulnerable to rapid clearance by local enzymes and the harsh oxidative microenvironment, hindering sustained therapeutic accumulation at the lesion site [23]. In addition, repeated needle punctures to maintain effective therapeutic concentrations risk aggravating mechanical damage to the AF and accelerating disc degeneration [24].

To address these delivery hurdles, injectable hydrogels have been widely investigated as localized biomaterial platforms for disc repair [25]. In particular, microenvironment‐responsive hydrogels have demonstrated capacity to attenuate degenerative cascades, including ROS‐responsive matrices formulated to deliver SLC7A11 modRNA for ferroptosis inhibition [26] or composite systems engineered to scavenge radicals and modulate inflammatory signaling in IVDD models [27]. By conformally filling tissue defects within the disc space, such in situ‐forming hydrogels provide immediate biomechanical support while serving as localized reservoirs that physically retain therapeutic nanovesicles, mitigating rapid washout and enzymatic degradation [28, 29]. Given that disc degeneration is accompanied by sustained tissue acidosis and elevated ROS accumulation, developing hydrogel carriers that reflect native ECM mechanics while undergoing responsive degradation under acidic and oxidative conditions offers a viable strategy for sustained, on‐demand cargo release [30].

In this study, CDNVs were isolated and characterized. To achieve their sustained and stimuli‐responsive delivery within the degenerated disc microenvironment, an injectable hydrogel (HAPOCS) was engineered through dynamic cross‐linking between adipic dihydrazide and phenylboronic acid‐grafted hyaluronic acid (HA‐ADH‐PBA) and oxidized chondroitin sulfate (OCS). As fundamental constituents of the native cartilaginous matrix, both hyaluronic acid (HA) and chondroitin sulfate (CS) backbones inherently contribute to modulating inflammatory cues, enabling the hydrogel to actively ameliorate the local milieu beyond passive mechanical space‐filling [31, 32]. Furthermore, the incorporation of acid‐labile Schiff base bonds and ROS‐cleavable phenylboronate ester linkages enables the hydrogel network to undergo microenvironment‐triggered degradation, achieving on‐demand release of encapsulated CDNVs under pathological conditions [33]. Systematic in vitro and in vivo evaluations demonstrated that the resulting composite system (HAPOCS@CDNVs) effectively scavenges intracellular ROS, attenuates NPC premature senescence and SASP‐associated inflammation, and supports ECM homeostasis, thereby retarding structural collapse in degenerative discs (Scheme 1). Overall, this work presents a promising, minimally invasive biomaterial strategy that integrates plant‐derived nanotherapeutics with a responsive carrier for intervertebral disc repair.

**SCHEME 1:**
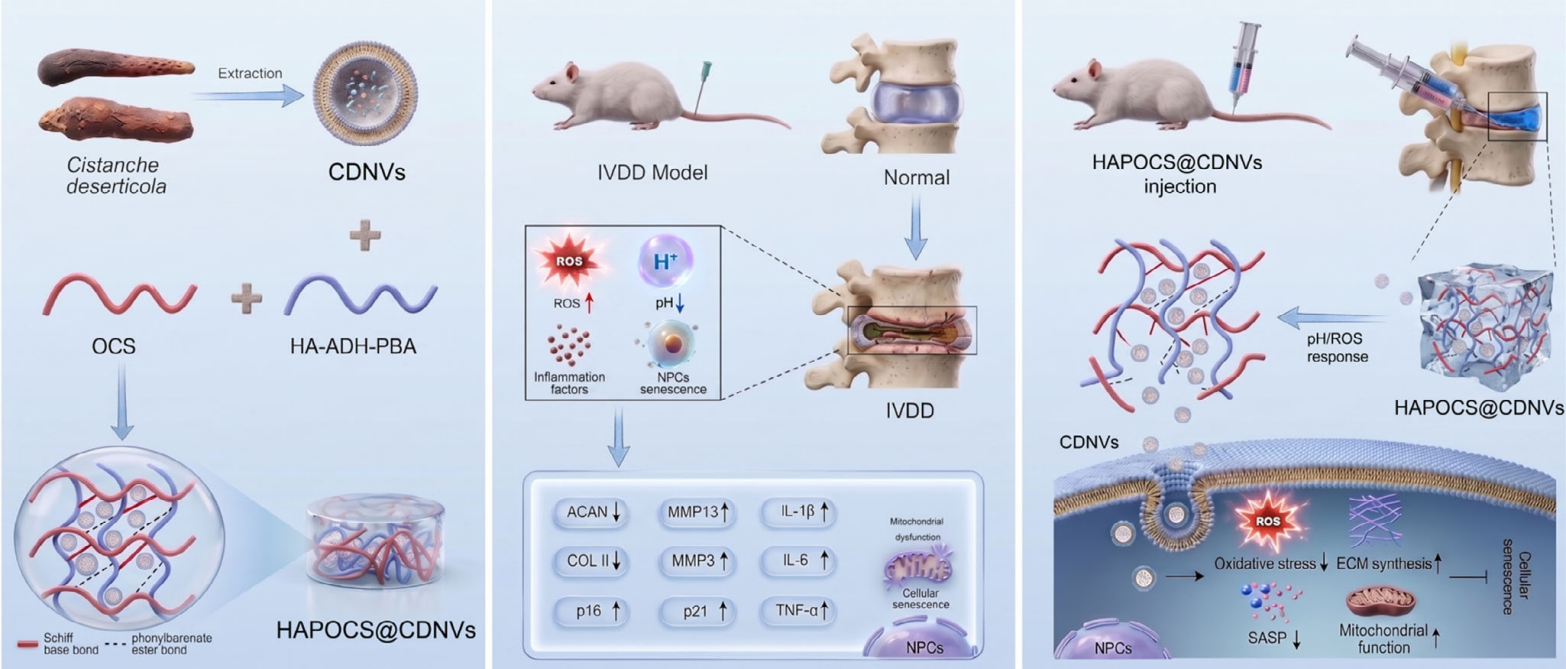
Schematic illustration of the fabrication and therapeutic mechanism of HAPOCS@CDNVs for intervertebral disc repair. The dual‐responsive hydrogel releases encapsulated CDNVs in response to local acidosis and elevated ROS levels, mitigating oxidative stress and cellular senescence while supporting ECM homeostasis to retard disc degeneration.

## 2 Results and Discussion

### 2.1 Isolation and Characterization of CDNVs

As a classic medicinal plant, specific extracts and core bioactive compounds of *Cistanche deserticola* have been widely reported to possess free radical‐scavenging and anti‐aging pharmacological activities. For instance, Wang et al. found that echinacoside, a primary active compound of C. deserticola, helps mitigate oxidative stress‐induced cellular ferroptosis and attenuate skeletal muscle atrophy by activating the IGF‐1/PI3K‐AKT pathway [34]. Furthermore, C. deserticola total glycosides have been shown to protect against cerebral ischemia‐reperfusion injury through antioxidant mechanisms involving the Nrf‐2/Keap‐1 signaling pathway [35]. However, traditional plant‐derived active compounds often face in vivo delivery bottlenecks, including low bioavailability, susceptibility to degradation in harsh microenvironments, and poor cellular uptake [36]. These limitations restrict their therapeutic potential. To overcome these barriers, we isolated CDNVs. As natural nanocarriers, PDNVs exhibit favorable biocompatibility and low immunogenicity. Their lipid bilayers protect internal cargos from rapid degradation, and they can be internalized by mammalian cells via endocytosis, facilitating intracellular delivery [37].

Differential ultracentrifugation combined with sucrose density gradient centrifugation is widely recognized as an established approach for isolating PDNVs [38]. Using this established approach, we processed fresh *Cistanche deserticola* tissues and successfully isolated CDNVs (Figure 1A). We then characterized these vesicles using transmission electron microscopy (TEM) and nanoparticle tracking analysis (NTA). TEM imaging revealed that the isolated CDNVs exhibited typical spherical or elliptical morphologies with intact lipid bilayer structures. Their diameters were predominantly under 500 nm, aligning with standard morphological features of PDNVs (Figure 1B) [39]. Quantitative NTA further demonstrated an average particle size of 147.2 nm (Figure 1C). The isolated CDNVs exhibited an average zeta potential of −38.27 ± 1.04 mV, indicating a negative surface charge that contributes to colloidal stability (Figure 1D). Subsequent fluorescent labeling with PKH67 showed that NPCs internalized the CDNVs in vitro (Figure 1E). Collectively, these findings indicate the isolation of structurally intact CDNVs capable of cellular uptake, establishing a basis for subsequent cargo profiling.

**FIGURE 1:**
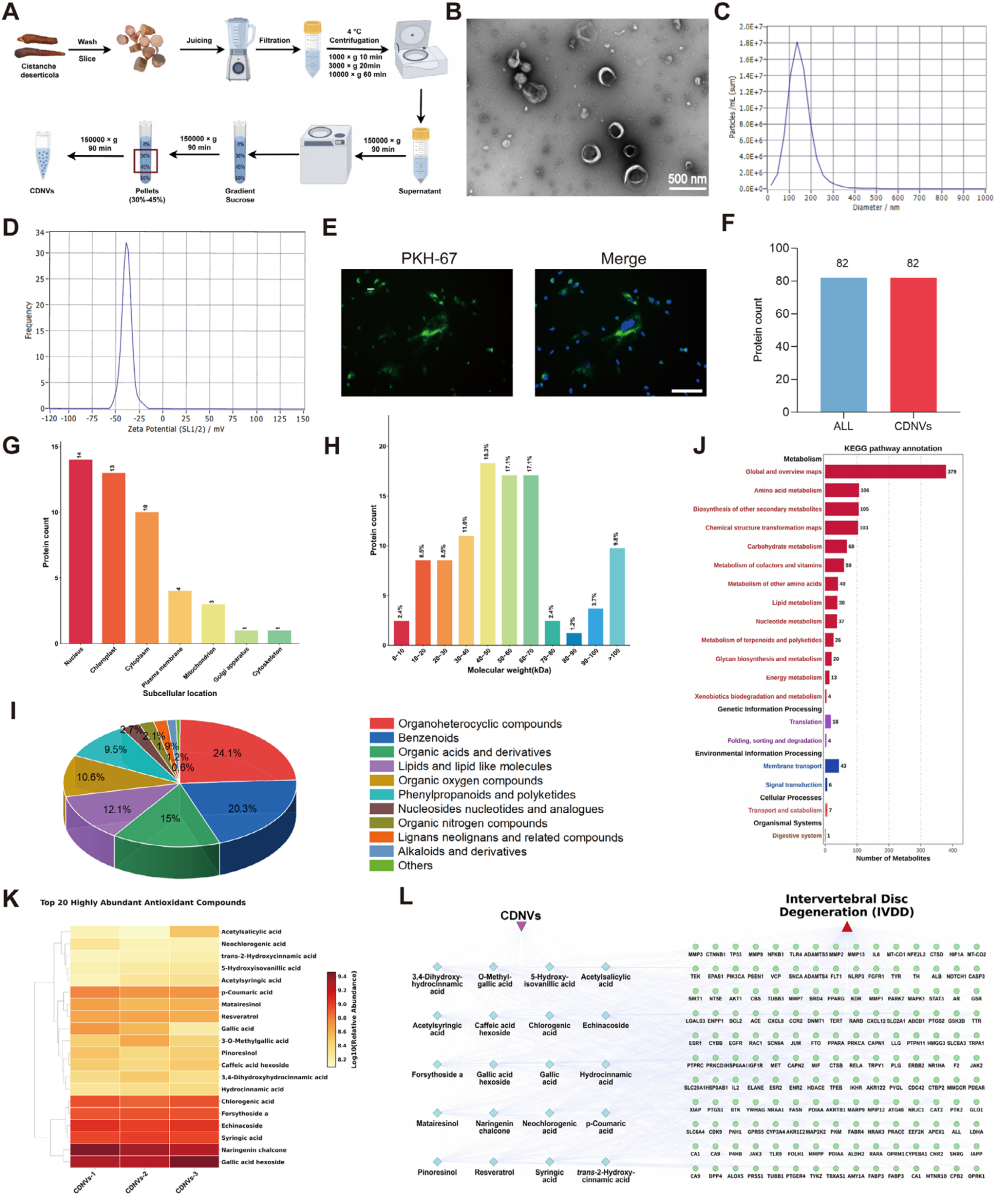
Isolation, characterization, and comprehensive omics profiling of CDNVs. (A) Schematic diagram shows the isolation process of CDNVs. (B) TEM evaluates the morphology and structure of the vesicles. (C) NTA measures the particle size distribution. (D) Zeta potential analysis detects the surface charge. (E) PKH67 fluorescence staining demonstrates the internalization of CDNVs by NPCs. Scale bar = 50 µm. (F) Proteomics analysis revealed the number of identified proteins. (G) Subcellular localization predicts the cellular origin of the proteins. (H) Proteomic analysis shows the molecular weight distribution of the identified proteins. (I) Metabolomic analysis calculates the classification and proportion of the chemical compounds. (J) KEGG analysis reveals the enriched metabolic pathways of the vesicle components. (K) Heatmap illustrating the relative abundance of the top 20 antioxidant compounds in CDNVs across three biological replicates. (L) The “Compound‐Target‐Disease” interaction network revealing the 20 core active compounds of CDNVs and their shared target genes with IVDD.

To explore their biological contents, preliminary proteomic profiling of the CDNVs identified 82 distinct proteins (Figure 1F). Subcellular localization predictions indicated that these proteins are distributed across various cellular compartments (Figure 1G). Molecular weight distribution analysis showed that approximately 85% of these proteins ranged from 0 to 80 kDa, while 15% were larger than 80 kDa (Figure 1H). Gene Ontology (GO) (Figure S1) and Kyoto Encyclopedia of Genes and Genomes (KEGG) pathway analyses (Figure S2) indicated that the identified proteins were primarily associated with cellular transport, macromolecular metabolism, and ATP binding. These data outline the compositional profile of the protein cargo identified in CDNVs. Notably, the presence of predicted nuclear and intracellular proteins in the proteomic profile reflects the intrinsic generation mechanism of PDNVs obtained via whole‐tissue processing [40]. Unlike apoplastic extracellular vesicles harvested non‐destructively via vacuum infiltration, CDNVs prepared through mechanical slicing and juicing are not generated solely through regulated exocytosis [41]. During mechanical tissue disruption, ruptured plasma and intracellular organelle membrane fragments rapidly re‐seal into vesicle‐like nanoparticles, yielding a heterogeneous population composed of secreted vesicles, intracellular vesicles, and reassembled membrane‐derived particles [42]. Consequently, while sucrose density‐gradient centrifugation effectively removes unencapsulated soluble proteins and non‐vesicular debris [43], intracellular and nuclear proteins entrapped within the re‐sealed lipid bilayers are retained as part of the composite nanovesicle cargo [44].

In addition to this macromolecular cargo, PDNVs contain diverse small‐molecule metabolites. Therefore, untargeted metabolomic profiling was performed to characterize these specific components. As illustrated in Figure 1I, the metabolomic profile delineates the chemical composition of CDNVs, dominated by organoheterocyclic compounds (24.1%) and benzenoids (20.3%), followed by organic acids and derivatives (15.0%), and lipids and lipid‐like molecules (12.1%). To examine these constituents, we further analyzed the detected classes of compounds.

Specifically, the vesicles contained several signature compounds, including phenylethanoid glycosides (e.g., echinacoside, salidroside, 6‐acetylacteoside, and forsythoside A) and iridoids (e.g., catalpol, geniposide, loganin, morroniside, and aucubin) characteristic of *Cistanche deserticola*, alongside detected phenolic acids, polyphenols, lignans, and flavonoids. Chemically, the presence of phenolic hydroxyl groups and oxygen‐containing heterocycles within these detected molecules suggests a potential capacity to act as electron donors capable of scavenging free radicals in vitro [45]. This chemical feature indicates that these constituents may contribute to the overall antioxidant profile of CDNVs. To further examine the annotation of CDNVs metabolites, KEGG pathway classification was performed (Figure 1J). The detected metabolites were broadly mapped to metabolic categories, including pathways associated with plant secondary metabolite biosynthesis, alongside annotations related to environmental information processing.

We subsequently analyzed the relative abundance of the top 20 annotated antioxidant‐related compounds in CDNVs. As shown in the clustering heatmap (Figure 1K), these 20 compounds were consistently detected across three independent replicates. Notably, Gallic acid hexoside and Naringenin chalcone exhibited higher relative abundance values (Log10 value > 9.2), followed by a cluster including Syringic acid, Echinacoside, Forsythoside a, and Chlorogenic acid. The detection of these recognized phenolic and flavonoid constituents indicates the presence of potential antioxidant‐related phytochemicals in CDNVs.

Finally, to explore potential target associations of CDNVs against IVDD, we constructed a “Compound‐Target‐Disease” interaction network (Figure 1L). This network identified 165 shared target genes between the 20 core compounds and IVDD. Based on these predicted targets, functional enrichment analysis suggests several potential pathways associated with ECM regulation (e.g., MMP2, MMP3, MMP9, MMP13, and the ADAMTS family), inflammatory responses (e.g., NFKB1, IL6, NLRP3, and PTGS2), oxidative stress, and cellular regulation (e.g., NFE2L2, SIRT1, TP53, BCL2, and CASP3). These predicted target and pathway associations provide testable hypotheses for future in vitro and in vivo studies.

### 2.2 CDNVs Alleviate Oxidative Stress, Inhibit Cellular Senescence, and Regulate Matrix Metabolic Balance in NPCs

During IVDD, the abnormal accumulation of ROS severely disrupts cellular redox equilibrium, and excessive ROS alongside damaged mitochondria constitute critical upstream drivers of cellular senescence [46]. Consequently, effectively scavenging intracellular ROS and rescuing mitochondrial function are imperative for mitigating IVDD progression. Prompted by metabolomic profiling that predicted potential antioxidant cargo in CDNVs, we investigated their cytoprotective efficacy against oxidative stress in NPCs exposed to H_2_O_2_.

To optimize the protective dosage of CDNVs against oxidative challenge, NPCs were treated with H_2_O_2_ alongside gradient concentrations of CDNVs. A Cell Counting Kit‐8 (CCK‐8) assay revealed that CDNVs dose‐dependently protected NPCs against H_2_O_2_‐induced cytotoxicity, with cell viability peaking at 50 µg/mL (Figure S3). Intracellular ROS levels were assessed using DCFH‐DA staining. H_2_O_2_ exposure triggered a robust intracellular ROS burst, whereas CDNV co‐treatment significantly attenuated this oxidative overload (Figure 2A and Figure S4A). Because mitochondria are central organelles susceptible to oxidative damage [47], we examined mitochondrial ultrastructure via TEM. Mitochondria in the H_2_O_2_ group exhibited evident swelling, vacuolization, and loss of cristae, whereas CDNV intervention preserved the structural integrity of the mitochondrial double membranes and cristae (Figure 2B). Mitochondrial membrane potential was further evaluated by JC‐1 staining. H_2_O_2_ induced pronounced mitochondrial depolarization, evidenced by a fluorescence shift from red J‐aggregates to green monomers; notably, CDNV treatment markedly reversed this transition, sustaining normal mitochondrial polarization (Figure 2C and Figure S4B). Furthermore, lipid peroxidation was monitored via C11‐BODIPY staining. The oxidative cascade initiated by H_2_O_2_ caused a distinct shift from red (unoxidized) to green (oxidized) fluorescence, which was substantially suppressed by CDNVs, maintaining the unoxidized red signal (Figure 2D and Figure S4C). Collectively, these findings demonstrate that CDNVs effectively alleviate H_2_O_2_‐induced oxidative stress in NPCs by scavenging intracellular ROS, maintaining mitochondrial structural and functional integrity, and inhibiting lipid peroxidation.

**FIGURE 2:**
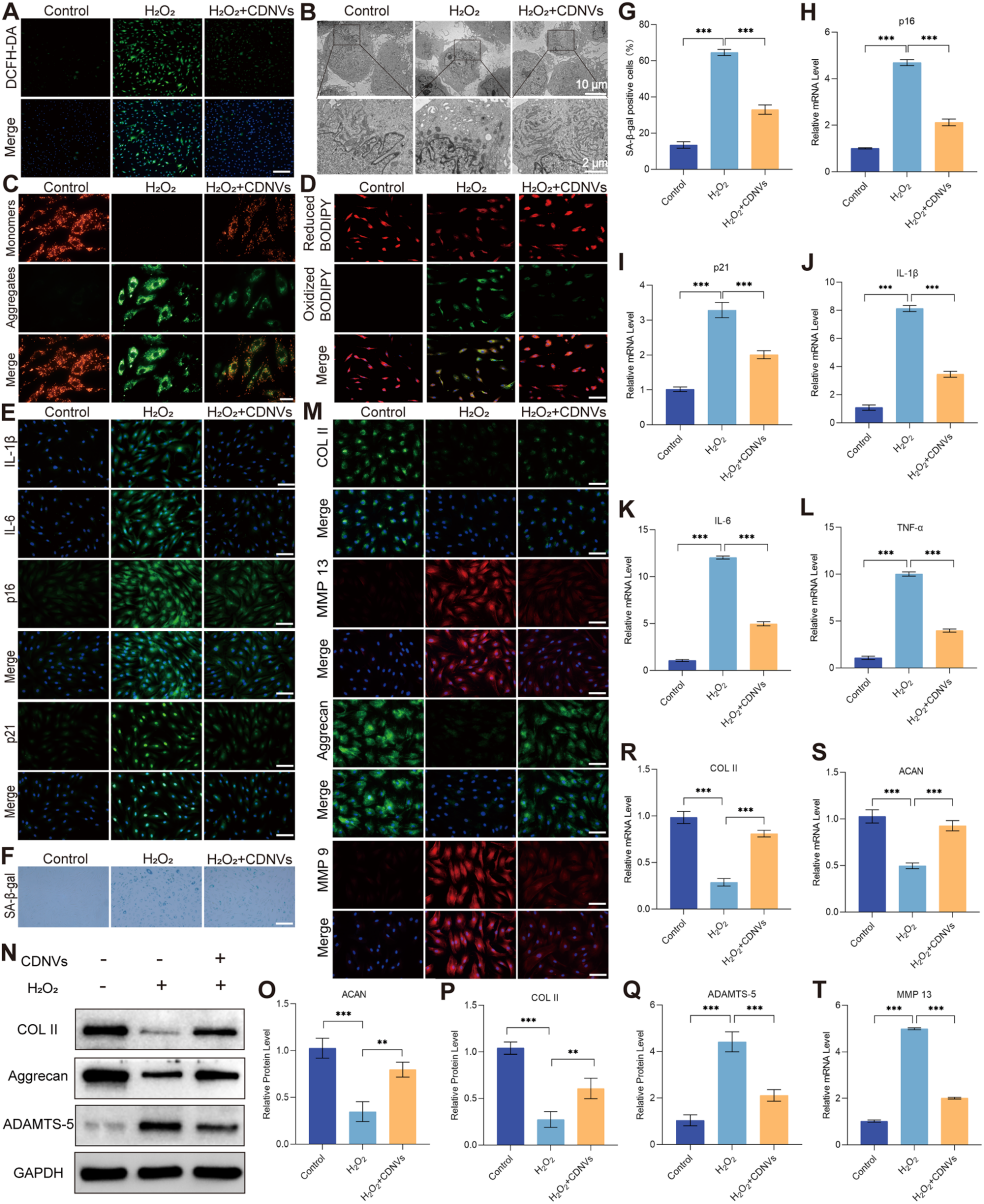
CDNVs alleviate oxidative stress, inhibit cellular senescence, and restore ECM homeostasis. (A) Representative DCFH‐DA fluorescence staining showing intracellular ROS levels. Scale bar = 100 µm. (B) Transmission electron microscopy (TEM) images showing mitochondrial structural integrity. Scale bar = 10 µm (top) and 2 µm (bottom). (C) JC‐1 staining evaluating mitochondrial membrane potential. Scale bar = 20 µm. (D) C11‐BODIPY staining assessing lipid peroxidation. Scale bar = 50 µm. (E) IF staining visualizes the expression of p16, p21, IL‐1β, and IL‐6. Scale bar = 50 µm. (F) SA‐β‐gal staining identifies senescent cells. Scale bar = 50 µm. (G) Statistical analysis quantifies the proportion of senescent cells (n = 3). (H‐L) RT‐qPCR analysis of the relative mRNA levels of p16, p21, IL‐1β, IL‐6, and TNF‐α (n = 3). (M) Representative IF staining images of COL II, MMP13, ACAN, and MMP9. Scale bar = 50 µm. (N) Western blot evaluates the protein expression of COL II, ACAN, and ADAMTS‐5. (O‐Q) Quantitative analysis of relative protein levels for (O) ACAN, (P) COL II, and (Q) ADAMTS‐5 normalized to GAPDH (n = 3). (R‐T) Relative mRNA expression levels of ECM markers detected by RT‐qPCR: (R) COL II, (S) ACAN, and (T) MMP13 (n = 3). Data are presented as mean ± SD (n = 3). ^*^ *p* < 0.05, ^**^ *p* < 0.01, ^***^ *p* < 0.001 by one‐way ANOVA with Tukey's multiple comparisons test.

We next evaluated whether CDNVs could alleviate H_2_O_2_‐induced senescence in NPCs. Senescent NPCs typically exhibit a SASP, characterized by the elevated secretion of pro‐inflammatory cytokines that accelerate ECM degradation [48]. Senescence‐associated β‐galactosidase (SA‐β‐gal) staining demonstrated that H_2_O_2_ exposure markedly increased the proportion of senescent cells, whereas CDNV treatment significantly reduced the percentage of SA‐β‐gal‐positive NPCs (Figure 2F,G). Immunofluorescence (IF) staining further confirmed that CDNVs suppressed the expression of classical senescence markers (p16 and p21) alongside key SASP factors (IL‐1β and IL‐6) (Figure 2E and Figure S5). Consistently, quantitative real‐time PCR (RT‐qPCR) revealed that CDNVs significantly downregulated the mRNA levels of these markers, as well as TNF‐α (Figure 2H–L). Together, these results indicate that CDNVs effectively alleviate stress‐induced cellular senescence in NPCs, thereby attenuating SASP factor release and interrupting the inflammatory cascade within the degenerative microenvironment.

The dynamic equilibrium between ECM synthesis and catabolism by NPCs is central to maintaining the physiological function of the intervertebral disc, while the cascade of oxidative stress, mitochondrial dysfunction, cellular senescence, and inflammation serves as a primary driver of matrix degradation [6]. In the H_2_O_2_‐induced in vitro oxidative stress model, CDNVs effectively alleviated matrix metabolic imbalance at both the protein and transcriptional levels. IF staining revealed that CDNV intervention markedly enhanced the protein abundance of core anabolic markers, type II collagen (COL II) and aggrecan (ACAN), while substantially suppressing the accumulation of catabolic enzymes (MMP13 and MMP9) (Figure 2M and Figure S6). Western blot and corresponding quantitative analysis further verified this protective effect at the protein level, confirming that CDNVs significantly restored the expression of COL II and ACAN, while counteracting the abnormal surge of the key catabolic enzyme ADAMTS‐5 (Figure 2N–Q). Consistent with these protein‐level changes, RT‐qPCR further demonstrated that CDNVs significantly upregulated the mRNA expression of COL2A1 and ACAN, alongside a marked downregulation of MMP13 (Figure 2R–T).

Together, these complementary molecular findings indicate that CDNVs scavenge excessive ROS and preserve mitochondrial integrity, which in turn attenuates stress‐induced cellular senescence and SASP secretion. By alleviating intracellular oxidative stress and reducing the release of pro‐inflammatory mediators, CDNVs effectively suppress matrix‐degrading enzymes while supporting the preservation of core structural proteins in NPCs. Collectively, these results provide important mechanistic insights into how CDNVs can ameliorate the pathological microenvironment of the degenerative intervertebral disc.

### 2.3 CDNVs Alleviate NPC Degeneration by Inhibiting NF‐κB Signaling

To investigate the transcriptomic alterations underlying the protective effects of CDNVs, we performed high‐throughput RNA sequencing (RNA‐seq) on NPCs across untreated Control, H_2_O_2_, and H_2_O_2_ + CDNVs groups. Hierarchical clustering analysis of a curated 15‐gene signature revealed that CDNV treatment effectively counteracted the pathological expression alterations induced by H_2_O_2_, partially restoring the overall transcriptomic profile toward the Control baseline (Figure 3A). Specifically, volcano plot analysis between the H_2_O_2_ and Control groups showed marked upregulation of catabolic matrix metalloproteinases (*MMP3*, *MMP9*, and *MMP13*) alongside pro‐inflammatory mediators (*IL1A*, *IL1B*, *IL6*, *IL32*, *CXCL1*, *CXCL5*, and *CXCL10*), accompanied by pronounced downregulation of core structural ECM components (*ACAN*, *COL11A1*, *COL26A1*, *COL9A2*, and *COL21A1*) (Figure 3B). Conversely, volcano plot comparison between the H_2_O_2_ + CDNVs and H_2_O_2_ groups confirmed that CDNV intervention significantly attenuated the activation of catabolic and inflammatory mediators while promoting the expression of key structural matrix transcripts (Figure 3C).

**FIGURE 3:**
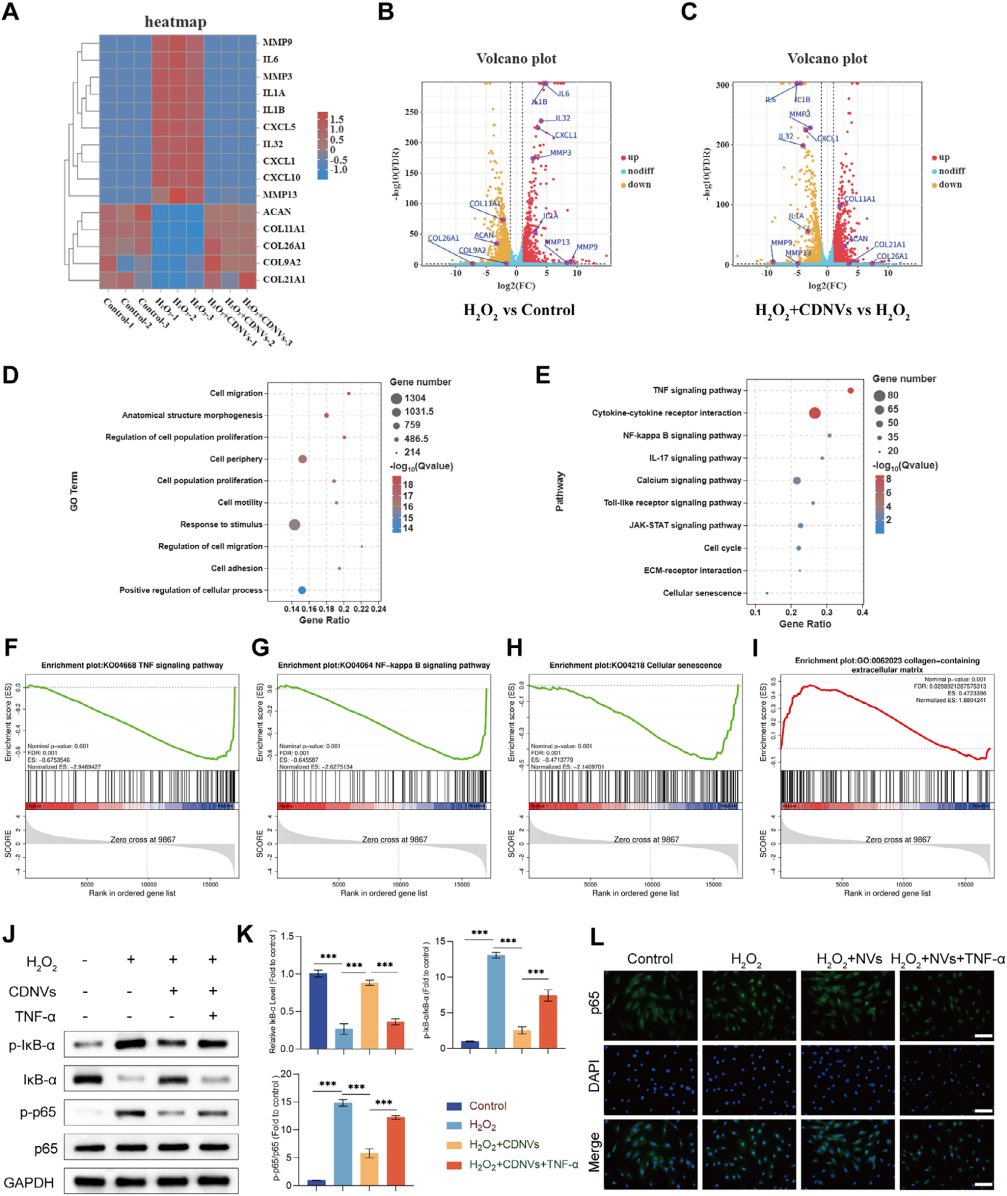
Transcriptomic characterization and experimental validation of CDNV‐mediated NF‐κB pathway inhibition. (A) RNA‐seq heatmap of 15 hallmark genes across Control, H_2_O_2_, and H_2_O_2_ + CDNVs groups (n = 3 biological replicates per group). (B, C) Volcano plots of differentially expressed genes (DEGs) in (B) H_2_O_2_ versus Control and (C) H_2_O_2_ + CDNVs versus H_2_O_2_. (D) GO biological process enrichment analysis of identified DEGs. (E) KEGG pathway enrichment analysis of identified DEGs. (F–I) Gene set enrichment analysis (GSEA) of (F) TNF signaling pathway, (G) NF‐κB signaling pathway, (H) cellular senescence, and (I) collagen‐containing ECM in the H_2_O_2_ + CDNVs versus H_2_O_2_ comparison. (J) Western blot analysis of p‐IκB‐α, IκB‐α, p‐p65, p65, and GAPDH across indicated treatment groups. (K) Densitometric quantification of total IκB‐α, p‐IκB‐α/IκB‐α, and p‐p65/p65 ratios (n = 3). Data are presented as mean ± SD; ^***^p < 0.001 by one‐way ANOVA followed by Tukey's post‐hoc test. (L) IF staining of p65 (green) counterstained with DAPI (blue). Scale bar = 50 µm.

To systematically elucidate the biological functions associated with these differentially expressed genes, GO enrichment analysis was conducted, which categorized the responsive genes into predominant biological processes, including response to stimulus, anatomical structure morphogenesis, cell motility, and cell adhesion (Figure 3D). Furthermore, KEGG pathway analysis revealed significant enrichment in degenerative and inflammatory signaling cascades, notably cytokine–cytokine receptor interaction, the TNF signaling pathway, the NF‐κB signaling pathway, and cellular senescence (Figure 3E). These pathway annotations directly align with the observed phenotypic rescue and suggest that modulation of inflammatory signaling may represent a primary mechanistic axis of CDNV action.

To further evaluate these alterations at the pathway level, we performed gene set enrichment analysis (GSEA) across both the modeling and treatment phases. Challenge with H_2_O_2_ elicited positive enrichment for the TNF signaling pathway (NES = 2.97, FDR = 0.001), the NF‐κB pathway (NES = 2.64, FDR = 0.001), and cellular senescence (NES = 1.81, FDR = 0.005), together with negative enrichment for the collagen‐containing ECM gene set (NES = −1.94, FDR = 0.008), consistent with the establishment of an inflammatory, catabolic state in vitro (Figure S7). Treatment with CDNVs yielded opposing enrichment trends across these pathways. In the H_2_O_2_ + CDNVs versus H_2_O_2_ comparison, gene sets for TNF signaling (NES = −2.95), NF‐κB signaling (NES = −2.63), and cellular senescence (NES = −2.14) were negatively enriched (all FDR = 0.001) (Figure 3F–H). Meanwhile, the ECM gene set displayed positive enrichment (NES = 1.88, FDR = 0.030) (Figure 3I). These reciprocal enrichment scores indicate that CDNVs suppress TNF, NF‐κB, and senescence‐associated pathways while supporting the expression of ECM components.

Across both KEGG and GSEA analyses, TNF and NF‐κB signaling emerged as prominent pathways activated by oxidative stress and suppressed by CDNVs. Because extracellular TNF drives inflammatory and catabolic cascades largely via downstream NF‐κB activation [49], we evaluated whether CDNVs protect NPCs by inhibiting this pathway. Western blotting revealed that H_2_O_2_ stimulated IκB‐α phosphorylation, promoted its degradation, and enhanced p65 phosphorylation. Treatment with CDNVs attenuated IκB‐α degradation and decreased both p‐IκB‐α/IκB‐α and p‐p65/p65 ratios. To examine functional dependence on this signaling cascade, we introduced recombinant TNF‐α as an exogenous pathway reactivator. Reactivation of the axis with TNF‐α counteracted the inhibitory effect of CDNVs on NF‐κB signaling, driving renewed IκB‐α degradation and elevated p65 phosphorylation (Figure 3J,K). IF staining verified the subcellular localization of p65 (Figure 3L). In the Control group, p65 was predominantly localized in the cytoplasm. Exposure to H_2_O_2_ induced marked nuclear translocation of p65, which was visibly suppressed by CDNV intervention, whereas exogenous TNF‐α promoted p65 nuclear re‐entry. Together, these results demonstrate that CDNVs attenuate oxidative degenerative responses in NPCs by blocking NF‐κB pathway activation through stabilizing IκB‐α and restricting p65 phosphorylation and nuclear translocation.

### 2.4 Fabrication, Characterization, and Biocompatibility of HAPOCS@CDNVs Hydrogels

Figure 4A outlines the design and synthetic pathway for the dynamic covalent hydrogel network. Because native NP tissue consists primarily of HA and CS‐rich proteoglycans, HA and CS were selected to mimic the biochemical composition of the ECM [50]. Beyond biocompatibility, the HA component provides targeted ligands for CD44 receptors on NPCs, promoting cellular adhesion and engaging downstream pathways that support matrix secretion while retarding dedifferentiation in degenerative environments [51]. Mixing HA‐ADH‐PBA and OCS aqueous solutions produced a self‐supporting hydrogel within minutes through dual dynamic covalent crosslinking (Figure 4B). This network relies on two reversible linkages: acylhydrazone bonds (a stable class of Schiff base linkages) formed via condensation between the dialdehyde groups of OCS and the hydrazide moieties of HA‐ADH, and cyclic phenylboronate ester bonds established between the PBA groups of HA‐ADH‐PBA and the diol motifs of OCS. The acylhydrazone linkages undergo protonation and accelerated hydrolysis in weakly acidic conditions (pH 5.5–6.5), rendering the matrix responsive to the local acidosis of degenerated discs [52]. Meanwhile, the carbon–boron bonds within the phenylboronate ester units undergo oxidative cleavage in the presence of ROS, providing an on‐demand dissociation mechanism coupled with local radical consumption [53].

**FIGURE 4:**
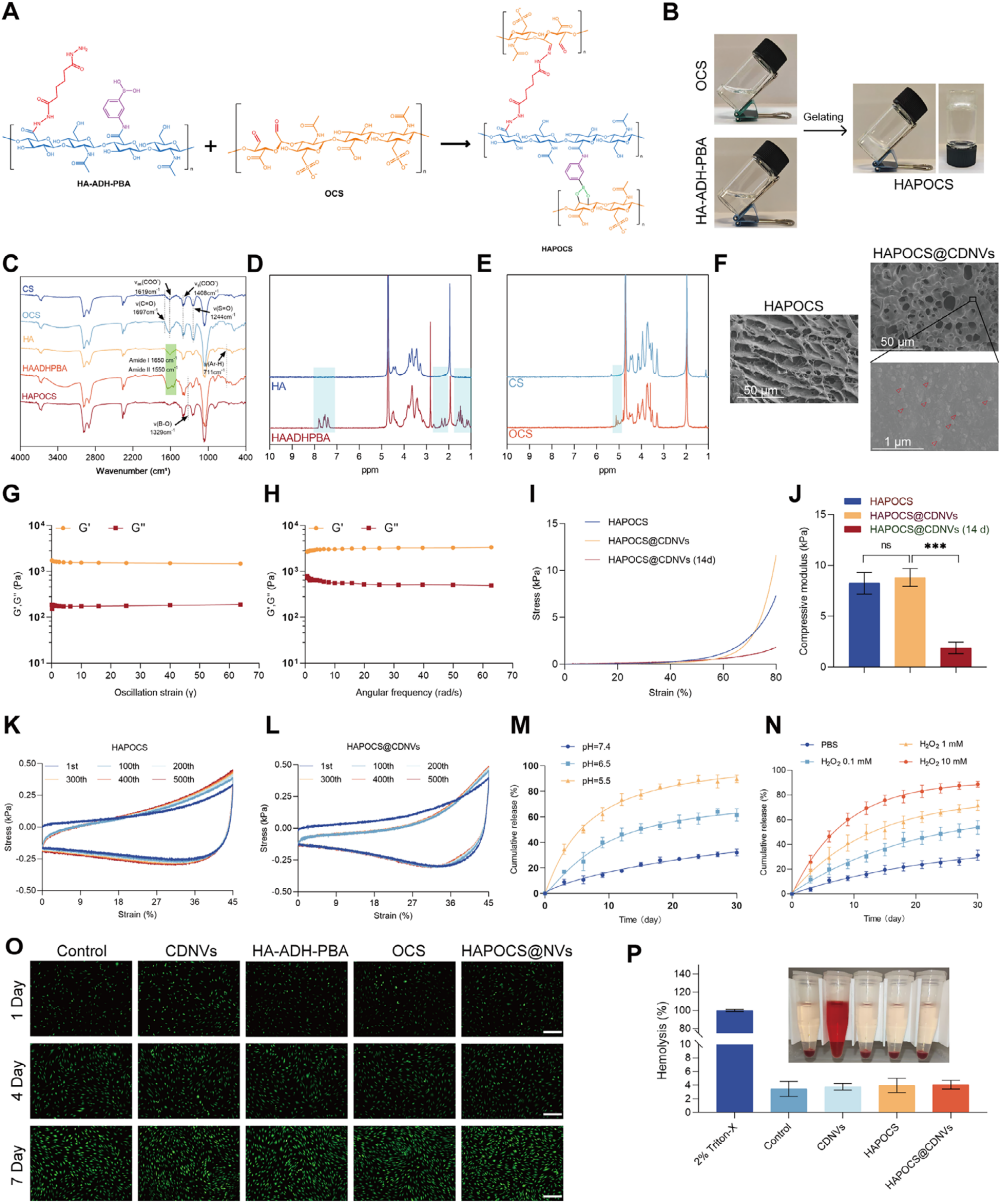
Fabrication, characterization, and biocompatibility of the HAPOCS@CDNVs hydrogel. (A) Schematic diagram illustrates the design and synthesis route of the dynamic covalent hydrogel. (B) Macroscopic photographs display the in situ formation of the hydrogel. (C) FT‐IR spectra verify the chemical structures and crosslinking bonds of the precursors and hydrogel. (D, E) ^1^H‐NMR spectra confirm the successful chemical synthesis of the polymers. (F) Scanning electron microscopy visualizes the internal porous morphology of the hydrogels. (G, H) Rheological analysis measures the storage and loss moduli to evaluate mechanical properties. (I) Uniaxial compressive stress–strain curves of blank HAPOCS, freshly prepared HAPOCS@CDNVs, and HAPOCS@CDNVs after 14 days of in vitro degradation. (J) Corresponding compressive moduli calculated from the linear elastic regime (n = 3). (K, L) 500‐cycle continuous compressive loading‐unloading hysteresis curves of HAPOCS (K) and HAPOCS@CDNVs (L) at 45% maximum strain, demonstrating fatigue resistance and superimposable loops. (M, N) In vitro release curves quantify the cumulative release rate of CDNVs under acidic and oxidative microenvironments. (O) Live/Dead staining evaluates the in vitro cytocompatibility of the hydrogels with NPCs. Scale bar = 100 µm. (P) Hemolysis assay assesses the blood compatibility of the hydrogels (n = 5). Data are presented as mean ± SD. ^*^ *p* <0.05, ^**^ *p* <0.01, ^***^ *p* <0.001; ns, not significant by one‐way ANOVA followed by Tukey's post‐hoc test.

Precursor modification and network crosslinking were characterized by Fourier transform infrared (FT‐IR) and proton nuclear magnetic resonance (^1^H‐NMR) spectroscopy. In the FT‐IR spectra, OCS exhibited a characteristic aldehyde C═O stretching band at 1697 cm^−^^1^, confirming the periodate‐mediated oxidation of CS, while HA‐ADH‐PBA displayed intensified amide I and II bands (∼1650 and ∼1550 cm^−^^1^) together with an aromatic C─H bending peak at 711 cm^−^^1^, verifying the conjugation of ADH and PBA onto the HA chain. Upon HAPOCS hydrogel formation, the disappearance of the 1697 cm^−^^1^ aldehyde peak and the attenuation of the B─O stretching band at 1329 cm^−^^1^ demonstrated the consumption of aldehyde and boronic acid groups to establish dynamic acylhydrazone and phenylboronate ester crosslinks (Figure 4C). ^1^H‐NMR spectra further corroborated the chemical structures of the modified precursors. For HA‐ADH‐PBA, along with the native HA backbone resonances (δ 2.0 ppm for the acetamido methyl protons and δ 3.0–4.0 ppm for the polysaccharide ring protons), newly emerging signals at δ 7.4–7.8 ppm (peaks 1–3, PBA aromatic ring) and δ 1.6 and 2.3–2.5 ppm (peaks 4 and 5, ADH methylene protons) confirmed the successful dual grafting (Figure 4D and Figure S8B). For OCS, the appearance of characteristic hemiacetal proton resonances at δ 5.0–5.2 ppm (peaks 1–2) resulting from aldehyde hydration verified the generation of dialdehyde units (Figure 4E and Figure S8A).

Scanning electron microscopy (SEM) imaging showed that both blank and CDNVs‐loaded hydrogels possessed interconnected porous structures, with high‐magnification views revealing CDNVs attached to the inner pore walls, indicating that vesicle loading preserved the porous architecture (Figure 4F). The viscoelastic behavior of the hydrogels was then evaluated by oscillatory rheology. In strain sweeps at 1 Hz, the storage modulus (G') exceeded the loss modulus (G'') across a wide linear viscoelastic regime (>60%), indicating elastic‐dominant gel behavior under shear deformation (Figure 4G). In frequency sweeps at fixed strains (0.5%, 1%, and 10%), G' remained consistently higher than G'' across all tested frequencies without crossover, reflecting a stable crosslinked network (Figure 4H and Figure S9A,B).

Uniaxial and cyclic compression tests were further performed to evaluate the load‐bearing capacity and fatigue resistance of the hydrogels. Uniaxial compression yielded nonlinear, J‐shaped stress–strain curves across all groups, with CDNVs incorporation increasing the compressive stress at 80% strain from 7.2 kPa (blank HAPOCS) to 11.3 kPa, suggesting a physical reinforcement effect at large deformations (Figure 4I). The initial compressive modulus of HAPOCS@CDNVs (9.6 ± 1.0 kPa) was comparable to that of blank HAPOCS (8.3 ± 1.0 kPa, *p* >0.05), indicating that vesicle loading maintained network stiffness; after 14 days of in vitro incubation (HAPOCS@CDNVs (14 d)), bond hydrolysis reduced the modulus to 1.9 ± 0.6 kPa (*p* <0.001), though the hydrogel retained its macroscopic shape (Figure 4J). During 500‐cycle compression at 45% strain, the slight negative stress upon early unloading resulted from wet interfacial adhesion and delayed detachment between the hydrogel and the platen; after the first cycle, hysteresis loops from the 100th to 500th cycle overlapped closely with minimal peak stress decay, indicating stable energy dissipation and fatigue resistance under repetitive loading (Figure 4K,L). Both the G' plateau (∼3 × 10^3^ Pa) and the compressive moduli fall within the reported range for native NP tissue [28, 54], suggesting suitable mechanical compatibility with the disc microenvironment.

In vitro release assays were conducted to evaluate CDNV‐associated protein release kinetics from the hydrogel under simulated pathological conditions. Under mildly acidic conditions (pH 5.5) mimicking local disc acidosis, acid‐triggered hydrolysis of the acylhydrazone bonds accelerated the cumulative release of CDNV‐derived protein components, reaching nearly 90% cumulative release over 30 days compared with slower release at neutral pH (Figure 4M). Similarly, in an oxidative environment simulated by 10 mM H_2_O_2_, ROS‐induced cleavage of the phenylboronate ester linkages drove cumulative release to approximately 90% over the same period (Figure 4N). This dual‐responsive behavior enables sustained, on‐demand cargo delivery in response to local acidic and oxidative cues. However, because release was quantified using a Micro‐BCA assay, these profiles reflect total CDNV‐associated protein kinetics rather than exclusive intact vesicle quantification, as protein assays cannot distinguish intact CDNVs from lysed fragments or free proteins. Thus, the current data serve as a macro‐kinetic indicator of hydrogel‐mediated delivery, while vesicle‐specific validation warrants further investigation.

Live/Dead staining showed that NPCs cultured with hydrogel extracts maintained high viability and proliferated over 1, 4, and 7 days across all groups, indicating good in vitro cytocompatibility (Figure 4O). In hemolysis assays, all material groups exhibited hemolysis rates below 5%, with values comparable to the saline negative control (Figure 4P). These findings demonstrate that the HAPOCS@CDNVs hydrogels possess favorable in vitro cytocompatibility and hemocompatibility.

### 2.5 In Vitro Attenuation of Oxidative Stress, Senescence, and Matrix Degradation by the HAPOCS@CDNVs Hydrogel

To evaluate the therapeutic efficacy of the hydrogel delivery system against oxidative stress‐induced NPC degeneration, an H_2_O_2_‐challenged cellular model was established. To investigate the protective effects of the sustained‐release CDNVs while preventing direct mechanical perturbation from the bulk hydrogel matrix, a non‐contact Transwell co‐culture system (0.4 µm pore size) was utilized. Specifically, H_2_O_2_‐treated NPCs were seeded in the lower chamber, whereas the HAPOCS@CDNVs hydrogel was placed in the upper insert. This physical separation design prevents direct cellular compression while enabling responsive released CDNVs to diffuse across the semipermeable membrane and exert their protective bioactivity on the damaged target cells below (Figure 5O).

**FIGURE 5:**
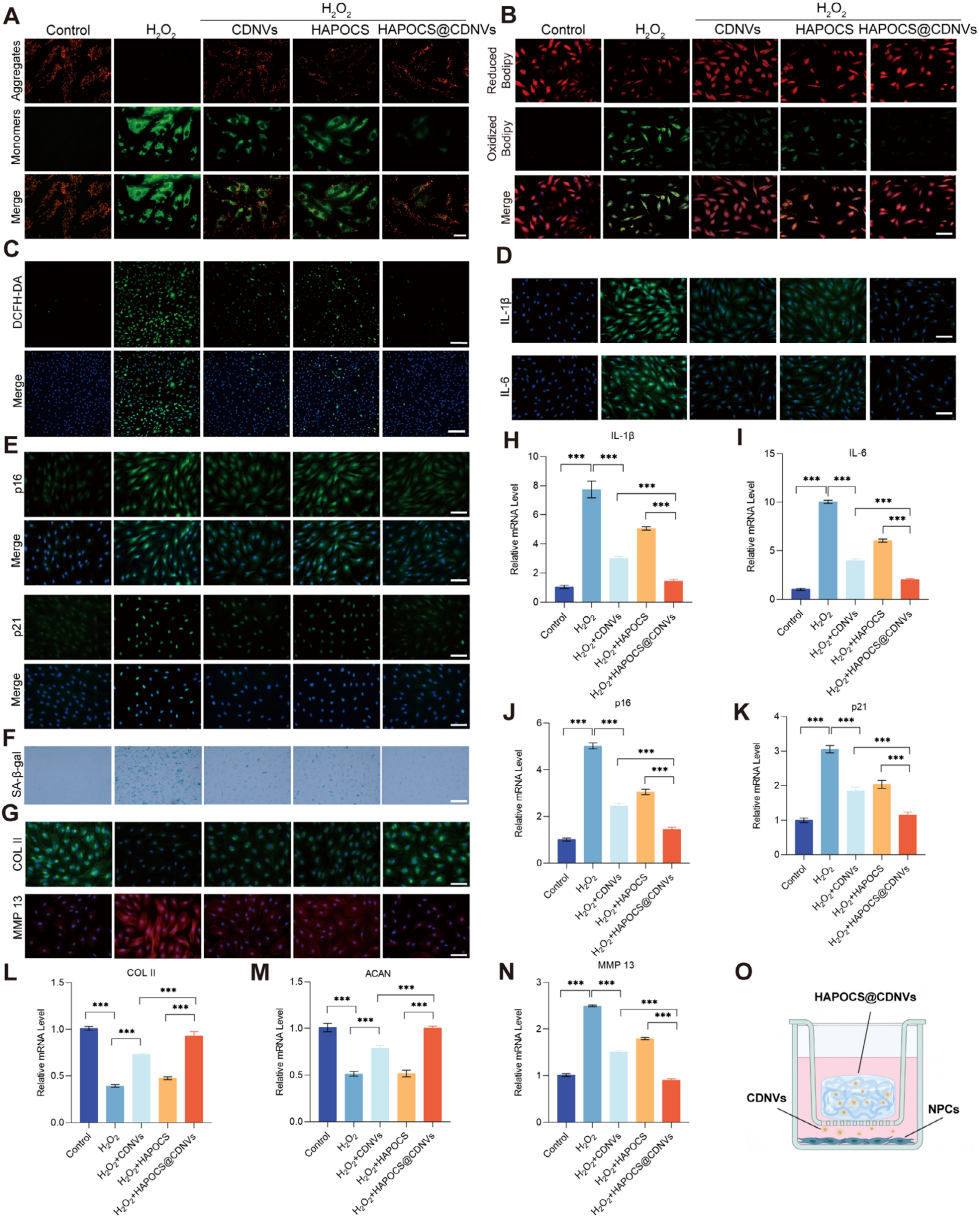
In vitro attenuation of oxidative stress, senescence, and matrix degradation by the HAPOCS@CDNVs hydrogel. (A) JC‐1 staining assesses mitochondrial membrane potential. Scale bar = 20 µm. (B) C11‐BODIPY staining quantifies the degree of lipid peroxidation. Scale bar = 50 µm. (C) DCFH‐DA staining measures total intracellular ROS levels. Scale bar = 100 µm. (D‐E) IF staining visualizes the protein expression of IL‐1β, IL‐6, p16, and p21. Scale bar = 50 µm. (F) SA‐β‐gal staining identifies senescent cells. Scale bar = 50 µm. (G) IF staining visualizes the protein expression of COL II and MMP13. Scale bar = 50 µm. (H‐K) RT‐qPCR detects the mRNA expression levels of IL‐1β, IL‐6, p16, and p21 (n = 3). (L‐N) RT‐qPCR detects the mRNA expression levels of COL II, ACAN, and MMP13 (n = 3). (O) Schematic diagram illustrates the Transwell non‐contact co‐culture system of the HAPOCS@CDNVs hydrogel and NPCs. Data are presented as mean ± SD. ^*^ *p* <0.05, ^**^ *p* <0.01, ^***^ *p* <0.001; ns, not significant by one‐way ANOVA followed by Tukey's post‐hoc test.

Using this co‐culture system, the mitochondrial membrane potential of NPCs was evaluated using a JC‐1 probe (Figure 5A and Figure S10A). Untreated control cells exhibited strong red fluorescence (J‐aggregates), characteristic of polarized mitochondria, whereas H_2_O_2_ exposure triggered a pronounced transition to green monomeric fluorescence, indicating mitochondrial depolarization. Treatment with CDNVs alone or the blank HAPOCS hydrogel partially attenuated this membrane depolarization. Notably, the HAPOCS@CDNVs hydrogel group exhibited the most pronounced restoration of mitochondrial polarization, as evidenced by a substantial recovery of red punctate fluorescence and a corresponding reduction in green monomers. Intracellular oxidative damage was further examined by evaluating lipid peroxidation via C11‐BODIPY staining (Figure 5B and Figure S10B) and total ROS levels via DCFH‐DA staining (Figure 5C and Figure S10C). While the individual treatments with CDNVs or the blank hydrogel moderately alleviated the oxidative burden induced by H_2_O_2_, the HAPOCS@CDNVs hydrogel significantly suppressed intracellular ROS accumulation and markedly attenuated lipid peroxidation (Figure 5B,C). These results indicate that the sustained release of CDNVs from the hydrogel network effectively mitigates intracellular oxidative stress, suppresses lipid peroxidation, and sustains mitochondrial membrane polarization in NPCs.

Furthermore, the anti‐inflammatory and anti‐senescent capacities of the treatments were investigated. IF (Figure 5D and Figure S11A,B) and RT‐qPCR (Figure 5H,I) analyses revealed that H_2_O_2_ stimulation significantly upregulated the expression of pro‐inflammatory cytokines IL‐1β and IL‐6, triggering a pronounced SASP. Concurrently, widespread positive SA‐β‐gal staining (Figure 5F and Figure S12A) and significantly elevated cell‐cycle arrest markers p16 and p21 (Figures 5E,J,K and S11C,D) indicated the pronounced induction of premature cellular senescence. Although CDNVs alone and the blank HAPOCS hydrogel provided moderate alleviation, the HAPOCS@CDNVs treatment exhibited the most pronounced reduction in these senescent markers and SASP‐related cytokines, significantly outperforming the individual treatments. Finally, the restoration of ECM homeostasis in NPCs was evaluated. IF (Figure 5G and Figure S12B,C) and RT‐qPCR (Figure 5L–N) results demonstrated that H_2_O_2_‐induced oxidative stress markedly suppressed matrix synthesis while promoting catabolism. In contrast, HAPOCS@CDNVs treatment significantly enhanced the expression of core anabolic components, including COL II and ACAN, while concurrently downregulating the matrix‐degrading enzyme MMP13, thereby mitigating the ECM metabolic imbalance in damaged NPCs.

The favorable therapeutic performance of the HAPOCS@CDNVs hydrogel system likely stems from the coordinated action between the functional polymeric matrix and the encapsulated vesicles. Specifically, the dynamic phenylboronate ester bonds within the network chemically consume ambient H_2_O_2_, enabling the hydrogel to act as an ROS scavenger that mitigates local oxidative stress [55]. Additionally, the hyaluronic acid and chondroitin sulfate backbones inherently contribute to an anti‐inflammatory microenvironment [56, 57]. When encapsulated within the network, the hydrogel serves as a protective physical reservoir and ROS‐scavenging barrier, providing stimuli‐responsive, sustained release that circumvents the rapid washout typical of unencapsulated CDNVs. Consequently, the ROS‐scavenging matrix acts cooperatively with the sustained bioactivity of the released CDNVs to facilitate the effective attenuation of NPC premature senescence, SASP‐associated inflammation, and ECM degradation.

### 2.6 In Vivo Structural Restoration and Microenvironmental Reprogramming by the HAPOCS@CDNVs Hydrogel

To evaluate in vivo delivery and therapeutic outcomes, we established a rat tail IVDD model by needle puncture (Figure 6A) [58]. We initially tracked the local retention kinetics of CDNVs to determine whether hydrogel encapsulation attenuated the rapid diffusion and clearance typical of unencapsulated vesicles. Longitudinal in vivo fluorescence imaging showed that free CDNVs cleared rapidly from the disc space, leaving minimal signal by day 7 and falling below the detection limit by day 14 (Figure 6B). In contrast, HAPOCS@CDNVs remained clearly visible locally throughout the 28‐day observation window. Quantitative retention kinetic analysis confirmed this extended profile: the HAPOCS hydrogel significantly prolonged the retention half‐life (t_1_/_2_) from 1.62 ± 0.04 days (free CDNVs) to 10.50 ± 0.31 days, yielding an approximately 4.8‐fold increase in the area under the curve (AUC_1__−__28_ d: 1131.9 ± 54.5 vs. 238.4 ± 2.1% days, *p* <0.001; Figure 6C).

**FIGURE 6:**
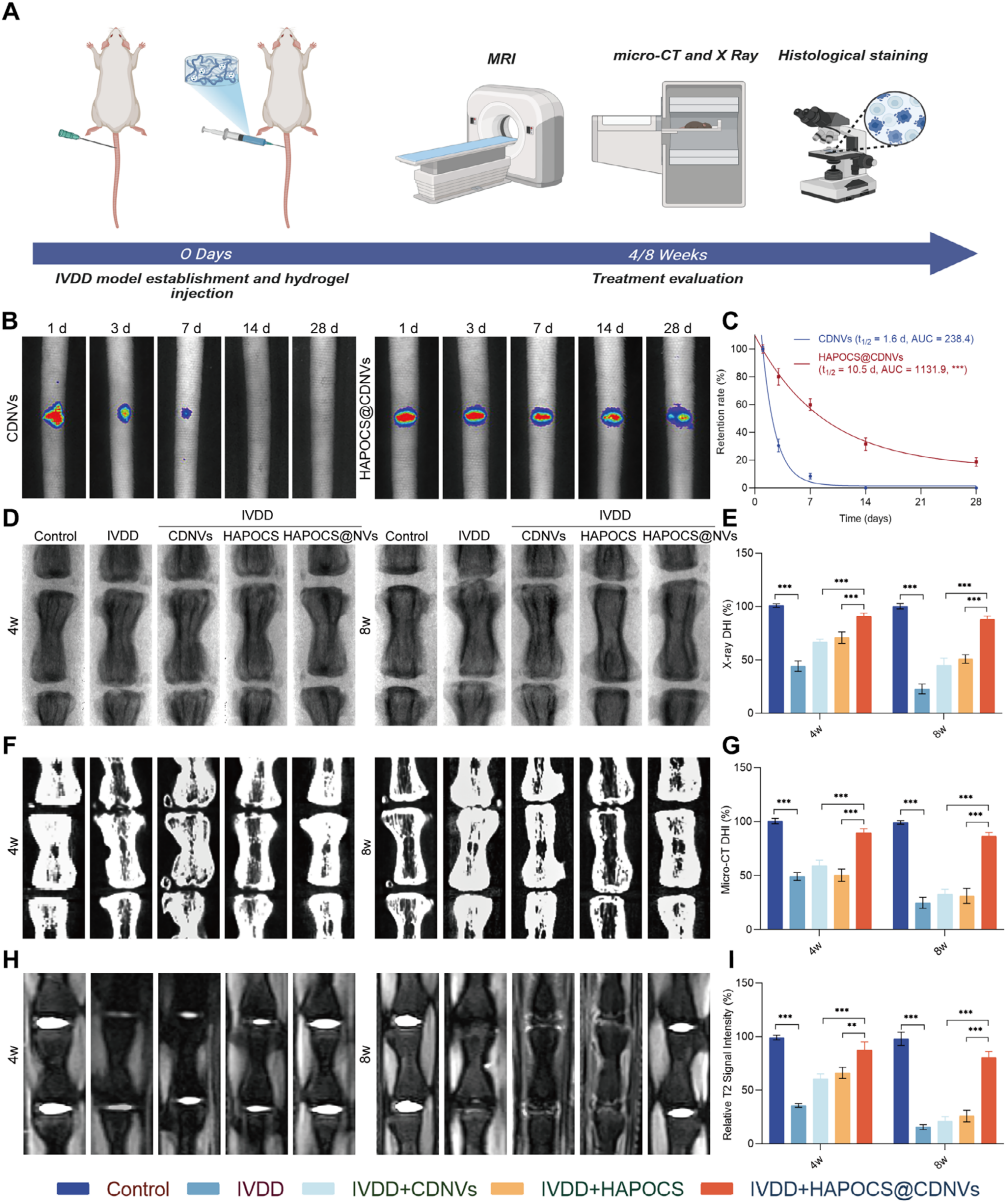
In vivo structural restoration and disc preservation by the HAPOCS@CDNVs hydrogel. (A) Schematic illustration of the rat tail IVDD needle‐puncture model establishment and the experimental timeline. (B) Representative longitudinal in vivo fluorescence tracking of rat tail discs at 1, 3, 7, 14, and 28 days post‐injection of free CDNVs or HAPOCS@CDNVs (n = 3). (C) In vivo local retention kinetics of free CDNVs and HAPOCS@CDNVs over 28 days (n = 3). Unencapsulated CDNVs underwent rapid clearance (t_1_/_2_ = 1.62 ± 0.04 days; AUC_1__−__28_ d = 238.4 ± 2.1% · days), whereas delivery via the HAPOCS hydrogel significantly prolonged the retention half‐life (t_1_/_2_ = 10.50 ± 0.31 days, *p* <0.001) and elevated cumulative local retention (AUC_1__−__28_ d = 1131.9 ± 54.5% · days, *p* <0.001). (D) Representative radiographic X‐ray images assessing intervertebral disc morphology and disc space height at 4 and 8 weeks post‐injection. (E) Quantitative assessment of the disc height index (DHI) calculated from X‐ray radiographs (n = 5). (F) Representative 3D micro‐CT reconstruction images evaluating intervertebral disc space and subchondral bone integrity at 4 and 8 weeks. (G) Quantitative evaluation of DHI calculated from micro‐CT scans (n = 5). (H) Representative sagittal T2‐weighted MRI images evaluating NP matrix hydration at 4 and 8 weeks post‐injection. (I) Quantification of relative T2 signal intensity in the NP (n = 5). Data are presented as mean ± SD. Statistical significance was determined by two‐tailed unpaired Student's t‐test (for two groups) or one‐way ANOVA followed by Tukey's post hoc test (for multiple comparisons). ^*^ *p* <0.05, ^**^ *p* <0.01, ^***^ *p* <0.001.

We then assessed structural preservation across the treated disc segments at 4 and 8 weeks post‐injection. The untreated IVDD discs developed progressive disc space narrowing and endplate sclerosis, as observed by both x‐ray (Figure 6D) and micro‐CT (Figure 6F). While treatment with free CDNVs or the blank HAPOCS hydrogel alone partially attenuated disc space collapse at 4 weeks, neither could sustain long‐term structural preservation by 8 weeks. Conversely, the HAPOCS@CDNVs hydrogel effectively preserved disc height and mitigated subchondral bone sclerosis throughout the 8‐week observation period. Consistent with these radiographic findings, the disc height index (DHI) quantified from both x‐ray (Figure 6E) and micro‐CT (Figure 6G) was significantly higher in the HAPOCS@CDNVs group than in the untreated IVDD and individual treatment groups at 8 weeks.

To monitor matrix hydration within the NP, sagittal T2‐weighted MRI was performed at 4 and 8 weeks post‐injection (Figure 6H). The hyperintense T2 signal within the NP decreased markedly in untreated IVDD discs by 4 weeks and was substantially diminished by 8 weeks, reflecting progressive matrix dehydration. Although individual treatment groups (free CDNVs or blank HAPOCS) retained moderate signal intensity at 4 weeks, this protective effect waned considerably by 8 weeks. In contrast, HAPOCS@CDNVs sustained significantly higher T2 signal intensity across both time points. Quantitative evaluation of relative T2 signal intensity (Figure 6I) confirmed that the composite hydrogel significantly mitigated NP dehydration compared to the untreated IVDD and single‐treatment groups. Together, these results indicate that delivery via the HAPOCS hydrogel effectively circumvents the rapid clearance of free CDNVs, providing sustained intra‐discal bioactivity to attenuate structural collapse and tissue degeneration in IVDD.

To evaluate in vivo tissue preservation and structural remodeling mediated by the hydrogel delivery system, histological assessments were performed on intervertebral disc specimens at 4 and 8 weeks post‐surgery. Safranin O‐Fast Green and hematoxylin and eosin (H&E) staining revealed that the untreated IVDD group exhibited marked structural disorganization and substantial glycosaminoglycan (GAG) loss at 4 weeks. By 8 weeks, degenerative changes were further aggravated, characterized by severe NP shrinkage and progressive fibrocartilaginous tissue ingrowth. While treatment with free CDNVs or the blank HAPOCS hydrogel alone provided modest structural protection at 4 weeks, this protective effect waned considerably by 8 weeks. In contrast, the HAPOCS@CDNVs hydrogel effectively sustained a well‐defined NP architecture and preserved intense GAG staining across both time points, demonstrating durable structural maintenance that approached the histological architecture of the Control group (Figure 7A,B).

**FIGURE 7:**
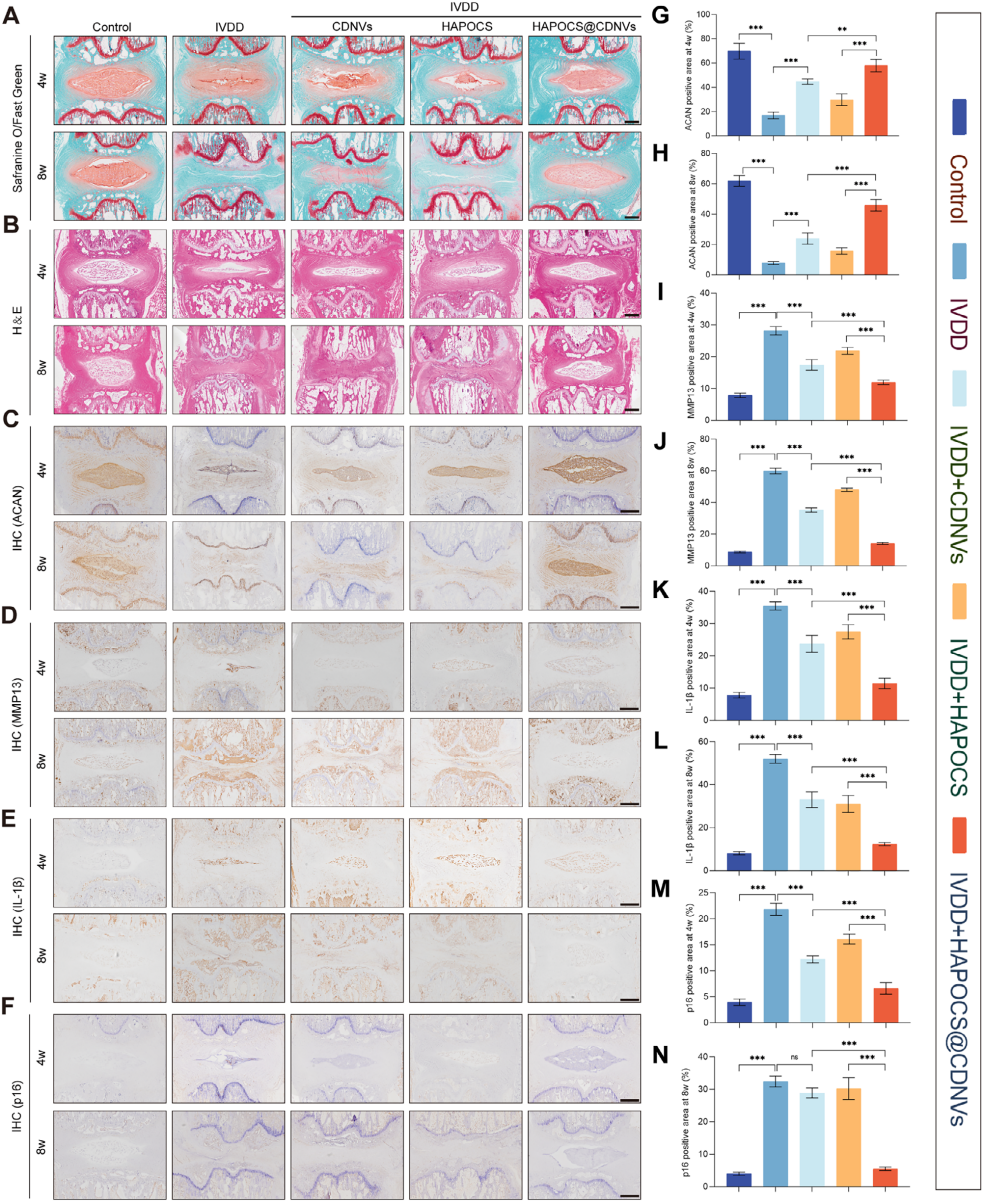
In vivo histological evaluation and microenvironmental reprogramming by the HAPOCS@CDNVs hydrogel. (A, B) Representative Safranin O–Fast Green (A) and H&E (B) staining evaluating the histological architecture and GAG distribution of rat tail intervertebral discs at 4 and 8 weeks post‐surgery. Scale bars = 500 µm. (C, D) Representative immunohistochemical staining evaluating the protein expression of the ECM anabolic marker ACAN (C) and catabolic enzyme MMP13 (D) in disc tissues. Scale bars = 500 µm. (E, F) Representative immunohistochemical staining assessing the protein levels of pro‐inflammatory cytokine IL‐1β (E) and cellular senescence marker p16 (F). Scale bars = 500 µm. (G–J) Quantitative analysis of relative positive areas for ACAN (G, H) and MMP13 (I, J) (n = 5). (K–N) Quantitative analysis of relative positive areas for IL‐1β (K, L) and p16 (M, N) (n = 5). Data are presented as mean ± SD. Statistical significance was determined by one‐way ANOVA followed by Tukey's post hoc test for multiple comparisons. ^*^ *p* <0.05, ^**^ *p* <0.01, ^***^ *p* <0.001; NS, not significant.

Subsequently, immunohistochemical analysis was performed to evaluate ECM metabolic equilibrium, as well as the sustained in vivo anti‐inflammatory and anti‐senescent effects of the hydrogel system. Qualitative staining and quantitative evaluations revealed progressive deterioration of the local microenvironment in the untreated IVDD group from 4 to 8 weeks. Specifically, the anabolic marker ACAN showed continuous depletion, whereas the matrix‐degrading enzyme MMP13 exhibited progressive accumulation (Figure 7C,D,G–J). Concurrently, the expression of the pro‐inflammatory cytokine IL‐1β and the cellular senescence marker p16 increased significantly over time (Figure 7E,F,K–N). While individual treatments with free CDNVs or the blank HAPOCS hydrogel provided moderate alleviation at 4 weeks, this protective effect waned by 8 weeks, likely due to the lack of a long‐term retention mechanism. In contrast, HAPOCS@CDNVs treatment not only significantly restored ACAN levels and suppressed MMP13 accumulation, but also markedly attenuated local inflammation and suppressed premature senescence in the degenerative disc (Figure 7C–N).

These protective effects were further corroborated at the spatial protein level via multiplex IF analysis. Consistent with the immunohistochemical observations, the HAPOCS@CDNVs group effectively sustained an organized ACAN fluorescent matrix throughout the 8‐week period, while substantially attenuating the abnormal accumulation of catabolic MMP3 (Figure 8A,C–F), as well as the senescence‐associated inflammatory factors IL‐6 and p16 (Figure 8B,G–J). Collectively, these complementary histological analyses indicate that the sustained delivery of CDNVs via the HAPOCS hydrogel effectively mitigates progressive IVDD by supporting matrix homeostasis, attenuating inflammation, and suppressing cellular senescence in vivo.

**FIGURE 8:**
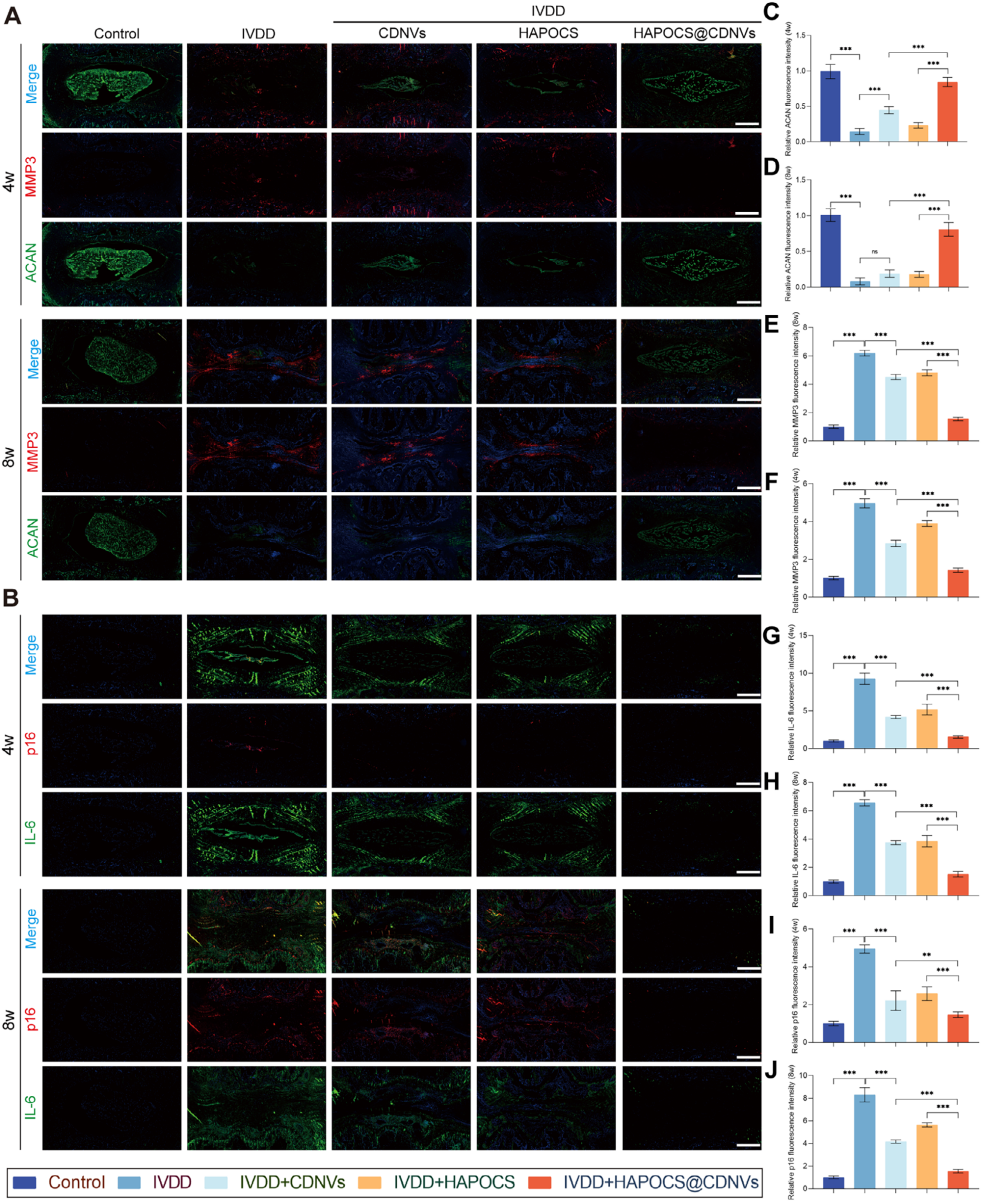
Multiplex IF analysis of ECM remodeling, inflammation, and cellular senescence in vivo. (A) Representative multiplex IF images showing the spatial protein distribution of ACAN and MMP3 in rat tail intervertebral disc tissues at 4 and 8 weeks post‐surgery. Scale bars = 500 µm. (B) Representative multiplex IF images showing the protein expression and localization of pro‐inflammatory cytokine IL‐6 and cellular senescence marker p16. Scale bars = 500 µm. (C–F) Quantitative evaluation of relative fluorescence intensity for ACAN (C, D) and MMP3 (E, F) (n = 5). (G–J) Quantitative evaluation of relative fluorescence intensity for IL‐6 (G, H) and p16 (I, J) (n = 5). Data are presented as mean ± SD. Statistical significance was determined by one‐way ANOVA followed by Tukey's post hoc test for multiple comparisons. ^*^ *p* <0.05, ^**^ *p* <0.01, ^***^ *p* <0.001; NS, not significant.

Beyond therapeutic efficacy, systemic biosafety is a fundamental prerequisite for the translational evaluation of such biomaterials. To evaluate in vivo biosafety at 4 weeks post‐implantation, histological examination of major organs (heart, liver, spleen, lungs, and kidneys) was conducted across the Control, CDNVs, HAPOCS, and HAPOCS@CDNVs groups, revealing no observable morphological abnormalities, structural damage, or adverse inflammatory cell infiltration (Figure S13). Furthermore, systemic evaluations across all five groups demonstrated that while untreated IVDD rats exhibited mild inflammatory stress, intervention with HAPOCS@CDNVs effectively normalized leukocyte differentials—including white blood cells (WBC), neutrophils, and lymphocytes—toward the baseline (Figure S14A–C). Meanwhile, erythrocyte indices (RBC, HGB) and platelets remained well within physiological ranges across all cohorts without signs of hematotoxicity (Figure S14D–F). In parallel, serum biochemical profiling confirmed that hepatic functional parameters (ALT, AST, ALP, ALB, and TP; Figure S15A–E) and renal metabolic indicators (CREA, BUN, and UA; Figure S15F–H) consistently resided within standard physiological limits, indicating negligible parenchymal injury or metabolic burden. Collectively, these findings substantiate the favorable in vivo biocompatibility and acceptable safety profile of the HAPOCS@CDNVs hydrogel.

The in vivo therapeutic outcomes revealed a distinct time‐dependent efficacy profile: while free CDNVs or the blank HAPOCS hydrogel provided moderate structural protection at 4 weeks, their benefits waned by 8 weeks. This decline is largely attributable to the rapid diffusion and clearance of unencapsulated CDNVs, as well as the lack of sustained bioactive cues in the blank hydrogel, despite its initial biomechanical buffering and ROS consumption. In contrast, the HAPOCS@CDNVs hydrogel effectively addresses these limitations by coupling biomechanical support with microenvironment‐responsive, sustained cargo release. Specifically, the resilient polymer network cushions dynamic spinal loading and protects encapsulated vesicles from rapid clearance, while the extended intra‐discal retention of CDNVs sustains NPC metabolic activity, attenuates cellular senescence, and suppresses SASP accumulation, thereby contributing to durable extracellular matrix preservation and disc hydration over 8 weeks.

While the HAPOCS@CDNVs hydrogel supported disc height retention and matrix replenishment over the 8‐week observation period, several translational and long‐term safety limitations should be acknowledged. Although systemic evaluations at 4 weeks indicated acceptable preliminary biocompatibility, with complete blood counts and hepatic/renal metabolic markers remaining within standard physiological ranges, a more exhaustive safety assessment is required prior to clinical translation. In particular, the in vivo metabolic clearance pathways and prolonged degradation kinetics of the hydrogel components were not directly tracked over extended timeframes. Additionally, while major organ histology showed no structural damage or adverse inflammatory infiltration, subtle micro‐inflammatory responses within the local disc space and potential reactions in adjacent structures, including the vertebral endplates and neighboring nerve roots, warrant deeper evaluation. Addressing these clearance kinetics and localized tissue interactions in larger animal models with extended follow‐up will help clarify the long‐term safety profile and translational feasibility of the HAPOCS@CDNVs hydrogel.

## 3 Conclusion

In this study, an injectable hydrogel (HAPOCS@CDNVs) crosslinked via dynamic Schiff base and phenylboronate ester bonds was constructed to achieve microenvironment‐responsive delivery of CDNVs. In vitro, CDNVs scavenged ROS, attenuated lipid peroxidation, and sustained mitochondrial membrane polarization, thereby inhibiting NF‐κB activation and downregulating senescence markers (p16, p21) and SASP factors (IL‐1β, IL‐6) in degenerate NPCs. This alleviation of oxidative stress and senescence subsequently restored matrix metabolism by upregulating COL II and ACAN while suppressing MMP3 and MMP13. In a rat puncture model, HAPOCS prolonged the intradiscal retention of CDNVs to 28 days. Over 8 weeks, intradiscal injection of HAPOCS@CDNVs maintained disc height and T2‐weighted MRI hydration signals, supported histological ACAN preservation, and reduced local MMP3, MMP13, IL‐1β, and p16 expression, without inducing histological or biochemical systemic toxicity. Together, these findings demonstrate that HAPOCS@CDNVs retards disc degeneration by mitigating oxidative stress, suppressing cellular senescence, and preserving ECM homeostasis, providing a practical, minimally invasive strategy for intervertebral disc repair.

## 4 Materials and Methods

### 4.1 Isolation of CDNVs 

Differential ultracentrifugation combined with sucrose density gradient centrifugation was utilized to isolate the CDNVs. Fresh *Cistanche deserticola* tissues were thoroughly washed, sliced, juiced, and filtered. The collected juice underwent sequential centrifugation at 4°C to remove impurities, starting at 1000 × g for 10 min, followed by 3000 × g for 20 min and 10 000 × g for 60 min. The resulting supernatant was then ultracentrifuged at 150 000 × g for 90 min using an ultracentrifuge (Hitachi, Tokyo, Japan) to collect the initial pellets. These pellets were loaded onto a sucrose density gradient consisting of 8%, 30%, 45%, and 60% layers. A final ultracentrifugation at 150 000 × g for 90 min was performed, and the purified vesicles were collected from the 30% to 45% gradient interface. Finally, the purified CDNVs were obtained and resuspended in sterile PBS for immediate use or storage at −80°C.

### 4.2 Transmission Electron Microscopy Analysis

The morphology and ultrastructure of the isolated CDNVs were characterized using transmission electron microscopy. Briefly, the purified vesicle suspension was diluted with phosphate‐buffered saline. A droplet of the diluted suspension was carefully placed onto a 200 mesh carbon‐coated copper grid and allowed to adsorb at room temperature for 5 min. Excess liquid was gently removed from the edge of the grid using filter paper. Subsequently, the sample was negatively stained with a 2% (w/v) uranyl acetate solution for 2 min in the dark. After removing the surplus staining solution with filter paper, the grid was air‐dried at room temperature. Finally, the morphological features and intact lipid bilayer structures of the CDNVs were visualized and photographed using a transmission electron microscope (Hitachi HT7800, Tokyo, Japan) operating at an accelerating voltage of 120 kV.

### 4.3 Zeta Potential Analysis

The surface charge and colloidal stability of the isolated CDNVs were evaluated by measuring the zeta potential. Briefly, the purified vesicle suspension was diluted with ultrapure water and carefully injected into a folded capillary cell without introducing air bubbles. The measurements were conducted at 25°C using a nanoparticle tracking analyzer (Particle Metrix ZetaView, Inning am Ammersee, Germany). All samples were measured in triplicate, and the final data were expressed as the mean zeta potential value with standard deviation.

### 4.4 Nanoparticle Tracking Analysis

The particle size distribution and concentration of the isolated CDNVs were determined using nanoparticle tracking analysis. Briefly, the purified vesicle suspension was diluted with phosphate‐buffered saline and injected into the sample chamber of a nanoparticle tracking analyzer (Particle Metrix ZetaView, Inning am Ammersee, Germany). The movement of the vesicles was recorded, and the data were analyzed to calculate the average particle size and concentration. All measurements were conducted in triplicate.

### 4.5 PKH67 Assay

The isolated CDNVs were labeled with a PKH67 green fluorescent cell linker kit (Sigma–Aldrich, St. Louis, MO, USA) to evaluate in vitro internalization. The vesicle suspension was mixed with the PKH67 dye solution and incubated at room temperature in the dark. Unbound dye was subsequently removed by ultracentrifugation, and the labeled vesicles were resuspended in phosphate‐buffered saline. NPCs were cultured in dishes and incubated with the PKH67‐labeled vesicles. Following the incubation period, the cells were washed with phosphate‐buffered saline and fixed with 4 percent paraformaldehyde. The cell nuclei were counterstained with DAPI (Solarbio, Beijing, China). Finally, the cells were washed with PBS and visualized using a laser scanning confocal microscope (Olympus, Tokyo, Japan).

### 4.6 Proteomic Profiling of CDNVs

Total proteins were extracted from the isolated CDNVs using radioimmunoprecipitation assay (RIPA) lysis buffer (Sigma–Aldrich, St. Louis, MO, USA) supplemented with protease inhibitors. The protein concentration was determined using a bicinchoninic acid (BCA) protein assay kit (Thermo Fisher Scientific, Waltham, MA, USA). The extracted proteins were reduced with dithiothreitol (DTT), alkylated with iodoacetamide (IAA), and digested overnight at 37°C using sequencing‐grade trypsin (Promega, Madison, WI, USA). The resulting peptide mixtures were desalted, dried, and resuspended in 0.1% formic acid. Liquid chromatography tandem mass spectrometry (LC‐MS/MS) analysis was performed on a Q Exactive mass spectrometer (Thermo Fisher Scientific) coupled with an Ultimate 3000 chromatography system (Thermo Fisher Scientific). The acquired raw mass spectrometry data were searched against the UniProt database for peptide and protein identification. Subcellular localization and molecular weight distribution of the identified proteins were predicted using bioinformatic tools. Finally, GO and KEGG pathway enrichment analyses were conducted to annotate the biological processes and functional pathways of the vesicular proteins.

### 4.7 Metabolomic Profiling of CDNVs

Metabolite extraction from the isolated CDNVs was performed using a pre‐cooled mixture of methanol, acetonitrile, and water (2:2:1, v/v/v) to precipitate proteins. The sample was vortexed, ultrasonicated in an ice bath, incubated at −20°C for 1 h, and centrifuged at 14 000 g for 20 min at 4°C. The supernatant was collected and evaporated to dryness using a vacuum concentrator (Eppendorf, Hamburg, Germany). The dried extracts were reconstituted in 50% acetonitrile and analyzed using a UHPLC system (Vanquish, Thermo Fisher Scientific) equipped with an amide column (Waters Corporation, Milford, MA, USA) coupled to a Q Exactive mass spectrometer (Thermo Fisher Scientific). Mass spectra were acquired in both positive and negative ionization modes using data‐dependent acquisition. Raw data were processed using Compound Discoverer software (Thermo Fisher Scientific) for peak picking and identification. Metabolite annotation was performed against the HMDB and mzCloud databases. Finally, metabolic pathways were identified via KEGG pathway enrichment analysis.

### 4.8 Cell Viability Assay

The cytoprotective effects of CDNVs against H_2_O_2_‐induced oxidative damage and the optimal intervention dosage were determined using a CCK‐8 assay (Beyotime Biotechnology, Shanghai, China). Briefly, NPCs were seeded into 96‐well culture plates at a density of 5 × 10^3^ cells per well and incubated overnight at 37°C with 5% CO_2_ to allow for cell attachment. Subsequently, the cells were exposed to H_2_O_2_ and co‐treated with varying concentrations of CDNVs in serum‐free medium for 24 h. Following the designated treatments, the culture medium was removed, and the cells were gently washed with PBS. Next, 100 µL of fresh culture medium containing 10 µL of CCK‐8 solution was added to each well. The plates were then incubated in the dark at 37°C for an additional 2 h. Finally, the optical density (OD) of each well was measured at an absorbance wavelength of 450 nm using a microplate reader (Thermo Fisher Scientific, Waltham, MA, USA). The relative cell viability was calculated as a percentage compared to the untreated control group. All experimental conditions were performed in triplicate.

### 4.9 Transwell Non‐Contact Co‐Culture System

A Transwell non‐contact co‐culture system (0.4 µm pore size, Corning, NY, USA) was utilized to evaluate the paracrine effects of CDNVs while avoiding mechanical compression from the hydrogel. NPCs were seeded in the lower chambers. After cell attachment, NPCs were treated with H_2_O_2_ to induce oxidative stress. Meanwhile, the blank HAPOCS hydrogel, HAPOCS@CDNVs hydrogel (containing 50 µg/mL CDNVs), or free CDNVs were added to the upper inserts. The inserts were then placed over the lower chambers, enabling the released CDNVs to freely diffuse across the membrane and interact with the damaged target cells. After co‐culturing for 24 h, the upper inserts were removed, and the NPCs in the lower chambers were subjected to subsequent downstream evaluations, including intracellular ROS measurement, IF staining, and RT‐qPCR.

### 4.10 Immunofluorescence Staining

The NPCs were purchased from Zhejiang Meisen Cell Technology Co., Ltd. (Zhejiang, China). The cells were cultured in DMEM/F12 medium supplemented with 10% fetal bovine serum (FBS) and 1% penicillin‐streptomycin. The cell cultures were maintained in a humidified incubator at 37°C with 5% CO_2_. NPCs seeded in confocal dishes were fixed with 4% paraformaldehyde (Sigma–Aldrich, St. Louis, MO, USA) for 15 min at room temperature and washed three times with PBS. Permeabilization was performed using 0.1% Triton X‐100 (Sigma–Aldrich) in PBS for 10 min, followed by three washes with PBS. Nonspecific binding sites were blocked with 5% bovine serum albumin (BSA; Sigma–Aldrich) in PBS for 1 h at room temperature. The cells were then incubated overnight at 4°C with primary antibodies diluted in 1% BSA, including anti‐COL II (28459‐1‐AP, Proteintech, China), anti‐ACAN (68350‐1‐Ig, Proteintech), anti‐MMP13 (83188–2‐RR, Proteintech), anti‐MMP9 (10375–2‐AP, Proteintech), anti‐p16 (ab270058, Abcam, UK), anti‐p21 (ab109520, Abcam), anti‐IL‐1β (26048–1‐AP, Proteintech), and anti‐IL‐6 (21865‐1‐AP, Proteintech). After washing three times with PBS, the cells were incubated with fluorophore‐conjugated secondary antibodies (Proteintech) in the dark for 1 h at room temperature. Nuclei were counterstained with DAPI for 5 min. Finally, the cells were washed with PBS and visualized using a laser scanning confocal microscope.

### 4.11 Western Blot Analysis

Total proteins were extracted from the treated cells using RIPA lysis buffer (Sigma–Aldrich, St. Louis, MO, USA) supplemented with protease and phosphatase inhibitor cocktails (Thermo Fisher Scientific, Waltham, MA, USA). Protein concentrations were quantified using a BCA protein assay kit (Thermo Fisher Scientific). Equal amounts of protein lysates (20 µg per lane) were separated by 10% sodium dodecyl sulfate‐polyacrylamide gel electrophoresis (SDS‐PAGE) and electro‐transferred onto polyvinylidene fluoride (PVDF) membranes (Millipore, Billerica, MA, USA). The membranes were blocked with 5% non‐fat milk in Tris‐buffered saline containing 0.1% Tween‐20 (TBST) for 1 h at room temperature to prevent nonspecific binding. Following blocking, the membranes were incubated overnight at 4°C with primary antibodies against COL II, ACAN, ADAMTS‐5 (ab41037, Abcam), and GAPDH (60004‐1‐Ig, Proteintech) as the internal loading control. After washing three times with TBST, the membranes were incubated with horseradish peroxidase (HRP)‐conjugated secondary antibodies (Proteintech) for 1 h at room temperature in the dark. The target protein bands were visualized using an enhanced chemiluminescence (ECL) substrate (Thermo Fisher Scientific) and captured by a ChemiDoc MP imaging system (Bio‐Rad, Hercules, CA, USA). The relative grey values of the protein bands were semi‐quantified using ImageJ software (National Institutes of Health, Bethesda, MD, USA) and normalized to the GAPDH reference bands.

### 4.12 RT‐qPCR Analysis

Total RNA was extracted from the treated NPCs using TRIzol reagent (Invitrogen, Carlsbad, CA, USA) according to the manufacturer's protocol. The purity and concentration of the isolated RNA were determined using a NanoDrop spectrophotometer (Thermo Fisher Scientific). For cDNA synthesis, equal amounts of total RNA (1 µg) were reverse transcribed using a PrimeScript RT reagent kit (Accurate Biological, China). RT‐qPCR amplification was performed on a LightCycler 480 Real‐Time PCR System (Roche Diagnostics, Basel, Switzerland) using SYBR Green Master Mix (Accurate Biological, China). The cycling conditions consisted of an initial denaturation at 95°C for 30 s, followed by 40 cycles of 95°C for 5 s and 60°C for 30 s. GAPDH was utilized as the internal reference gene. All primer sequences (Homo sapiens) used in this study are listed in Table S1. The relative mRNA expression levels were calculated using the comparative threshold cycle (2^−ΔΔCt^) method.

### 4.13 Intracellular ROS Assessment

Intracellular ROS levels within the treated NPCs were evaluated using a DCFH‐DA fluorescent probe kit (Beyotime Biotechnology, Shanghai, China). Following the respective experimental treatments, the cells cultured in multi well plates were gently washed with phosphate‐buffered saline. The cells were then incubated with the DCFH‐DA working solution diluted in serum‐free culture medium at 37°C for 20 min in the dark. After the incubation period, the cells were washed three times with serum‐free medium to completely remove the residual extracellular probe. The intracellular green fluorescence signals indicating ROS accumulation were immediately visualized and photographed using a laser scanning confocal microscope (Olympus, Tokyo, Japan). For quantitative comparison, the relative fluorescence intensity of the captured images was analyzed using ImageJ software.

### 4.14 Mitochondrial Membrane Potential Assessment

The mitochondrial membrane potential of the treated NPCs was evaluated using a JC‐1 fluorescent probe (Beyotime Biotechnology, Shanghai, China). The cells were washed with PBS and incubated with the JC‐1 working solution at 37°C for 20 min in the dark. After washing twice with the provided staining buffer, the cells were immediately observed and photographed under a laser scanning confocal microscope (Olympus, Tokyo, Japan). The mitochondrial membrane potential was determined by calculating the ratio of red to green fluorescence intensity using ImageJ software.

### 4.15 Lipid Peroxidation Assessment

The degree of lipid peroxidation in the treated NPCs was determined using a C11‐BODIPY 581/591 fluorescent probe (Thermo Fisher Scientific, Waltham, MA, USA). Following the designated treatments, the cells were incubated with 10 µM C11‐BODIPY in serum‐free culture medium at 37°C for 30 min in the dark. After the incubation period, the cells were washed three times with PBS to remove the extracellular probe. The shift of fluorescence from red to green, which indicates lipid peroxidation, was immediately observed and photographed using a laser scanning confocal microscope (Olympus, Tokyo, Japan). The relative levels of lipid peroxidation were quantified by calculating the ratio of green to red fluorescence intensity using ImageJ software.

### 4.16 Cellular Senescence Assessment

Cellular senescence was evaluated using a senescence‐associated β‐galactosidase (SA‐β‐gal) staining kit (Beyotime Biotechnology, Shanghai, China). The treated NPCs were washed with PBS and fixed with the provided fixative solution for 15 min at room temperature. After washing with PBS, the cells were incubated with the SA‐β‐gal working solution at 37°C overnight in an incubator without CO_2_. The senescent cells exhibiting blue staining were observed and photographed using a bright‐field microscope (Leica Microsystems, Wetzlar, Germany). The proportion of SA‐β‐gal positive cells was quantitatively analyzed to assess the degree of cellular senescence.

### 4.17 Transcriptomic Sequencing and Analysis

Total RNA was extracted from the treated NPCs using a TRIzol reagent (Invitrogen, Carlsbad, CA, USA). The integrity and concentration of the RNA were evaluated using an Agilent 2100 Bioanalyzer (Agilent Technologies, Santa Clara, CA, USA). For library construction, mRNA was enriched using mRNA Capture Beads and purified, followed by thermal fragmentation at an elevated temperature. The fragmented mRNA was then utilized as a template to synthesize the first strand cDNA within a reverse transcriptase mix system. During the synthesis of the second strand cDNA, end repair and A‐tailing were simultaneously performed. Subsequently, sequencing adapters were ligated, and the target fragments were purified and selected using Hieff NGS DNA Selection Beads (Yeasen Biotechnology, Shanghai, China). PCR amplification was then conducted to construct the library. Finally, high‐throughput sequencing was performed on an Illumina NovaSeq X Plus platform (Illumina, San Diego, CA, USA). Differential expression analysis, alongside GO and KEGG pathway enrichment analyses, was conducted to evaluate the transcriptomic variations.

### 4.18 Synthesis of OCS

Oxidized chondroitin sulfate (OCS) was synthesized via a sodium periodate oxidation method. Briefly, chondroitin sulfate (Sigma–Aldrich, St. Louis, MO, USA) was completely dissolved in ultrapure water. An aqueous solution of sodium periodate (NaIO_4_; Sigma–Aldrich) was added dropwise into the mixture and stirred continuously in the dark at room temperature for 24 h. To terminate the oxidation reaction, ethylene glycol was added and stirred for an additional 2 h. The resulting solution was dialyzed (molecular weight cutoff: 3500 Da) against ultrapure water for 3 days to remove unreacted reagents, and ultimately lyophilized to obtain the OCS powder.

### 4.19 Synthesis of HA‐ADH‐PBA

The HA‐ADH‐PBA conjugate was synthesized via a two‐step reaction process. In the first step, hyaluronic acid (HA; Sigma–Aldrich, St. Louis, MO, USA) was dissolved in ultrapure water, followed by the addition of adipic dihydrazide (ADH; Sigma–Aldrich) and 1‐ethyl‐3‐(3‐dimethylaminopropyl)carbodiimide hydrochloride (EDC; Sigma–Aldrich) to graft amino groups onto the HA backbone. The pH of the mixture was maintained at 4.8 overnight at room temperature. The intermediate product (HA‐ADH) was purified through extensive dialysis (molecular weight cutoff: 14 000 Da) against ultrapure water and subsequent lyophilization.

In the second step, the dried HA‐ADH was re‐dissolved in ultrapure water. 3‐aminophenylboronic acid (m‐APBA; Sigma–Aldrich) was added to the solution, and the reaction was initiated by adding EDC and N‐hydroxysuccinimide (NHS; Sigma–Aldrich) to conjugate the phenylboronic acid groups via amide bonds. The reaction mixture was stirred at room temperature for 24 h. The final HA‐ADH‐PBA product was purified by dialyzing against ultrapure water for 3 days and was ultimately obtained as a white spongy powder after freeze‐drying.

### 4.20 In Situ Administration of HAPOCS@CDNVs Hydrogel

To achieve in situ gelation and facilitate precise administration, a dual‐chamber syringe system equipped with a static mixing tip was utilized for the preparation and injection of the HAPOCS@CDNVs hydrogel. Briefly, the lyophilized HA‐ADH‐PBA polymer was dissolved in sterile PBS (pH 7.4) to a final concentration of 3% (w/v). The purified CDNV suspension was then uniformly dispersed into this solution to formulate Component A. Concurrently, the OCS polymer was dissolved in sterile PBS at 3% (w/v) to serve as Component B. Component A and Component B were subsequently loaded into the separate barrels of the dual‐chamber syringe. During the administration process, the two precursor solutions were co‐extruded through the static mixer, enabling instantaneous and homogeneous mixing. Driven by the rapid dynamic covalent crosslinking between the acylhydrazone and boronate ester bonds, the mixed precursor solutions immediately transitioned into a stable HAPOCS@CDNVs hydrogel network in situ at the targeted defect site.

### 4.21 FT‐IR Analysis

The chemical structures of the synthesized polymers and the freeze‐dried hydrogels were characterized using an FT‐IR spectrometer (Thermo Fisher Scientific, Waltham, MA, USA). Prior to the measurement, the lyophilized samples were uniformly ground with spectroscopic grade potassium bromide (KBr) powder and pressed into transparent pellets. The FT‐IR spectra were recorded at room temperature in the wavenumber range of 4000 to 400 cm^−^^1^ with a resolution of 4 cm^−^^1^ and 32 co‐added scans. The background spectrum of a blank KBr pellet was subtracted from each sample spectrum. The characteristic absorption peaks were subsequently analyzed to verify the successful synthesis and chemical crosslinking of the hydrogel networks.

### 4.22 ^1^H‐NMR Spectroscopy

The chemical structures of the synthesized macromolecules were further confirmed using a ^1^H‐NMR spectrometer (Bruker, Billerica, MA, USA) operating at 400 MHz. Briefly, the lyophilized polymer samples were completely dissolved in deuterium oxide (D2O; Sigma–Aldrich, St. Louis, MO, USA) at a concentration of approximately 10 mg/mL. All spectra were recorded at room temperature. The obtained free induction decay signals were Fourier‐transformed, and the spectral data were processed and analyzed using MestReNova software (Mestrelab Research, Santiago de Compostela, Spain) to verify the successful conjugation of the targeted functional groups.

### 4.23 SEM Analysis

The surface and cross‐sectional morphologies of the lyophilized hydrogels were characterized using an SEM (Hitachi, Tokyo, Japan). Prior to observation, the completely freeze‐dried hydrogel samples were carefully fractured to expose their internal porous structures. The fractured specimens were securely mounted onto aluminum stubs using double‐sided conductive carbon tape. To enhance electrical conductivity and prevent charge accumulation during imaging, the samples were sputter‐coated with a thin layer of gold for 60 s using an ion sputter coater. The micro‐morphologies were subsequently examined and photographed under the SEM at an accelerating voltage of 5.0 kV.

### 4.24 Rheological Characterization

The mechanical properties of the hydrogels were evaluated using a rotational rheometer (Anton Paar, Graz, Austria) equipped with a 20 mm parallel plate geometry. The testing gap was set to 1.0 mm, and the temperature was maintained at 37°C. To evaluate the viscoelastic behavior and identify the linear viscoelastic region, a strain amplitude sweep test was initially performed at a constant frequency of 1.0 Hz. Subsequently, frequency sweep tests were conducted over a frequency range of 0.1 to 100 rad/s. Specifically, these frequency sweeps were carried out under three different constant strains (0.5%, 1.0%, and 10%) to comprehensively monitor the storage modulus (G') and loss modulus (G''), thereby verifying the structural stability of the three‐dimensional crosslinked network under varying deformation conditions.

### 4.25 Mechanical Characterization

The compressive mechanical properties of the hydrogels were evaluated using a universal mechanical testing machine (Instron 5944, Norwood, MA, USA) equipped with a 50 N load cell. For unconfined uniaxial compression tests, cylindrical hydrogel specimens (8 mm in diameter, 9 mm in height) of HAPOCS, HAPOCS@CDNVs, and HAPOCS@CDNVs subjected to prolonged incubation were evaluated. For the 14‐day degradation group, HAPOCS@CDNVs hydrogels were immersed in PBS (pH 7.4, containing 0.02% w/v sodium azide to prevent microbial growth) and incubated at 37°C with gentle agitation (60 rpm), with the buffer refreshed every 48 h. Prior to testing, samples were gently blotted with filter paper to remove excess surface moisture. Uniaxial compression was performed up to a maximum strain of 80% at a constant crosshead speed of 5 mm/min. The compressive modulus was calculated from the slope of the linear elastic region within the 10%–20% strain range.

To assess fatigue resistance and energy dissipation, continuous cyclic loading–unloading tests were performed on cylindrical specimens of HAPOCS and HAPOCS@CDNVs for 500 consecutive cycles at a maximum strain of 45% with a constant crosshead speed of 5 mm/min. Hysteresis loops and peak stress evolution across the cycles were recorded to determine cyclic endurance and dynamic network recovery. All measurements were conducted with three independent replicates (n = 3).

### 4.26 In Vitro Release of CDNV‐Associated Proteins

The in vitro release profiles of CDNV‐associated proteins from HAPOCS@CDNVs hydrogels were evaluated under varying pH (7.4, 6.5, and 5.5) and ROS conditions (0, 0.1, 1.0, and 10 mM H_2_O_2_ in pH 7.4 PBS). Briefly, hydrogel samples (1.0 mL) were immersed in 3.0 mL of the respective release media and incubated at 37°C with continuous shaking at 100 rpm. At predetermined time intervals over a 30‐day period, 0.5 mL of the supernatant was collected and immediately replaced with an equal volume of fresh medium. The concentration of released CDNV‐associated proteins was quantified using a Micro BCA Protein Assay Kit (Thermo Fisher Scientific) according to the manufacturer's instructions to determine the cumulative release percentage of CDNV‐associated proteins.

### 4.27 Live/Dead Cell Viability Assay

The cytocompatibility of the hydrogels was evaluated using a Live/Dead Viability Kit (Beyotime, Shanghai, China). Cells were cultured with the hydrogel samples for 1, 4, and 7 days, followed by staining with Calcein‐AM and PI for 30 min in the dark. The cell morphologies were observed via fluorescence microscopy, and cell viability was quantified using ImageJ software.

### 4.28 In Vitro Hemolysis Assay

Hemocompatibility was assessed using a 2% (v/v) red blood cell suspension. Hydrogel samples were incubated with the suspension at 37°C for 2 h. PBS and 2% Triton X‐100 were used as negative and positive controls, respectively. After centrifugation, the absorbance of the supernatant was measured at 540 nm to calculate the hemolysis ratio.

### 4.29 Establishment of IVDD Model and in Vivo Treatments

All experimental procedures were conducted in strict accordance with ethical guidelines and were approved by the Institutional Ethics Committee of the First Affiliated Hospital of Xinjiang Medical University (No. 250303–77). An IVDD model was established in male Sprague‐Dawley rats (300 ± 20 g) by needle puncture. Briefly, the rats were anesthetized via intraperitoneal injection of tribromoethanol. The caudal discs (Co7/8 and Co8/9) were punctured using a 22‐gauge needle inserted into the disc center, rotated 360°, and held for 30 s. Immediately after puncture, 10 µL of sterile PBS (untreated IVDD group), free CDNVs, blank HAPOCS hydrogel, or HAPOCS@CDNVs hydrogel precursors was injected into the corresponding degenerated discs using a microsyringe. The concentration of CDNVs in both the free CDNVs group and the HAPOCS@CDNVs hydrogel group was 50 µg/mL. Post‐surgery, the injection sites were disinfected, and the rats were placed on a heating pad until complete recovery from anesthesia. Prophylactic antibiotics were administered for three consecutive days to prevent infection. The rats were housed in a pathogen‐free environment with ad libitum access to standard food and water. At 4 and 8 weeks post‐treatment, the rats underwent radiological evaluations, including X‐ray and micro‐CT imaging to assess the structural morphology and disc height index (DHI), as well as T2‐weighted MRI to evaluate the hydration status of the NP. Following imaging, the corresponding intervertebral disc tissues were harvested for subsequent evaluations.

### 4.30 In Vivo Retention Kinetics of CDNVs

CDNVs were fluorescently labeled with DiR (Biotium, USA) and washed by centrifugation to remove unbound dye. Following needle‐puncture IVDD modeling in rats, free DiR‐CDNVs or DiR‐labeled HAPOCS@CDNVs were injected into the Co7/8 disc space (n = 3 per group). Longitudinal in vivo fluorescence was tracked at days 1, 3, 7, 14, and 28 post‐injection using an IVIS Spectrum system (PerkinElmer; excitation/emission: 745/800 nm) under isoflurane anesthesia. Regions of interest (ROIs) were outlined in Living Image software to quantify radiant efficiency, with signals at each time point normalized to the day 1 baseline. Retention half‐life (*t*_1/2_) and area under the curve (AUC_1–28 d_) were determined via one‐phase decay fitting and trapezoidal integration, respectively.

### 4.31 Histological, Immunohistochemical, and Immunofluorescence Analyses

The harvested intervertebral disc specimens were fixed in 4% paraformaldehyde, decalcified in 10% EDTA, embedded in paraffin, and sectioned at a thickness of 5 µm. Prior to staining, all sections were deparaffinized in xylene and rehydrated through a graded ethanol series. For routine histological evaluation, the sections were stained with H&E and Safranin O‐Fast Green kits (Solarbio, Beijing, China) to observe general tissue morphology and GAG distribution. For immunohistochemical (IHC) and IF analyses, the sections underwent heat‐induced antigen retrieval and were blocked with 5% bovine serum albumin (BSA; Solarbio, Beijing, China). Subsequently, they were incubated overnight at 4°C with primary antibodies against ACAN, MMP13, MMP3, IL‐1β, IL‐6, and p16. For IHC, the sections were then incubated with HRP‐conjugated secondary antibodies, visualized using a DAB substrate kit (Servicebio, Wuhan, China), and counterstained with hematoxylin. For IF, the sections were incubated with corresponding fluorophore‐conjugated secondary antibodies (Servicebio, Wuhan, China), and cell nuclei were counterstained with DAPI (Solarbio, Beijing, China). Finally, all stained sections were observed under a microscope (Olympus, Tokyo, Japan), and the positive staining areas or fluorescence intensities were quantitatively analyzed using ImageJ software (Bethesda, MD, USA).

### 4.32 Statistical Analysis

The data were expressed as mean ± standard deviation (SD). Statistical analyses were conducted using GraphPad Prism 8.0 software (La Jolla, CA, USA). One‐way analysis of variance (ANOVA) combined with Tukey's multiple comparison test was used for comparison between groups. The significance of the differences between the two groups was determined using a two‐sided unpaired t‐test. Statistical significance was indicated by *p* <0.05. Symbols ^*^ *p* <0.05, ^**^ *p* <0.01, ^***^ *p* <0.001, and NS denoted no statistical significance.

## Author Contributions

**Ning Xu**: Conceptualization, Methodology, Writing – original draft. **Xiaoyu Cai**: Investigation, Formal analysis, Data curation, Funding acquisition. **Xiaofu Tang**: Validation, Visualization, Writing – original draft. **Weidong Liang**: Software, Validation. **Linsong Teng**: Investigation, Methodology. **Yudong Jing**: Formal analysis. **Haifeng Hu**: Resources, Investigation, Funding acquisition. **Yuhao Duan**: Validation, Data curation. **Mingjie Kuang**: Visualization, Formal analysis, Funding acquisition. **Lei Zhang**: Supervision, Writing –review & editing. **Shijie Han**: Project administration, Conceptualization, Supervision. **Weibin Sheng**: Conceptualization, Supervision, Resources, Writing – review & editing.

## Ethics Statement

All experimental procedures were conducted in strict accordance with ethical guidelines and were approved by the Institutional Ethics Committee of the First Affiliated Hospital of Xinjiang Medical University (No. 250303–77).

## Conflicts of Interest

The authors declare no conflicts of interest.

## Supporting information




**Supporting File**: advs78698‐sup‐0001‐SuppMat.docx.

## Data Availability

The data that support the findings of this study are available on request from the corresponding author.
