# Supplementary material for: Microenvironment‐Responsive Biomimetic Hydrogels Delivering Cistanche Deserticola‐Derived Nanovesicles for Intervertebral Disc Degeneration
Source: Adv Sci (Weinh). 2026 Nov 9:e78698. Online ahead of print. doi: 10.1002/advs.78698 (PMC13749007; doi:10.1002/advs.78698)
Supplement: Supplementary file 1 [file ADVS-9999-e78698-s001.docx]

**Supporting Information**

**Microenvironment-Responsive Biomimetic Hydrogels Delivering *Cistanche deserticola*-Derived Nanovesicles for Intervertebral Disc Degeneration**

Ning Xu^1†^, Xiaoyu Cai^1†^, Xiaofu Tang^2†^, Weidong Liang^1^, Lin-Song Teng^4^, Yudong Jing^5^, Haifeng Hu^2^, Yuhao Duan^2^, Mingjie Kuang^2^, Lei Zhang^3^*, Shijie Han^2^*, Weibin Sheng^1^*

1. Department of Spine Surgery, The First Affiliated Hospital of Xinjiang Medical University, Urumqi, 830013, P.R. China.
2. Department of Orthopedics, Provincial Hospital Affiliated to Shandong First Medical University, Jinan, 250021, P.R. China.
3. Department of Orthopedic Surgery, The First Affiliated Hospital of Shandong First Medical University & Shandong Provincial Qianfoshan Hospital, Tissue Engineering Laboratory, Department of Radiology, Shandong First Medical University, Jinan 250014, P.R. China.
4. Second Clinical Medical College of Shandong University of Traditional Chinese Medicine, Jinan, 250001, P.R. China.
5. Department of Nuclear Medicine, The First Affiliated Hospital of Shandong First Medical University & Shandong Provincial Qianfoshan Hospital, Jinan 250014, P.R. China.

^†^These authors contributed equally: Ning Xu, Xiaoyu Cai and Xiaofu Tang.

Corresponding Authors

Lei Zhang, E-mail: [qygkzl1818@163.com;](mailto:qygkzl1818@163.com;) Shijie Han, E-mail: [hanshijie@sdfmu.edu.cn;](mailto:hanshijie@sdfmu.edu.cn;) Weibin Sheng, E-mail: shengwb123123@163.com

**Figure S1**. Gene Ontology (GO) enrichment analysis of identified protein cargoes derived from Cistanche deserticola-derived nanovesicles (CDNVs).

**Figure S2.** Kyoto Encyclopedia of Genes and Genomes (KEGG) pathway analysis of the CDNV proteome.

**Figure S3.** Dose-dependent cytoprotective effects of CDNVs. The viability of cells under H₂O₂-induced oxidative stress was assessed via CCK-8 assay after treatment with different concentrations of CDNVs (0–100 μg/mL). Data are expressed as mean ± SD (n = 3).

**Figure S4.** (A) Relative fluorescence intensity of ROS fluorescent staining (n=3). (B) Quantification of the JC-1 red/green fluorescence ratio across different treatment groups (n=3). (C) Quantitative analysis of lipid peroxidation levels, represented by the fluorescence intensity ratio of oxidized (green) to reduced (red) C11-BODIPY (n=3). Data are presented as mean ± SD. *p < 0.05, **p < 0.01, ***p < 0.001; ns, not significant by one-way ANOVA followed by Tukey's post-hoc test.

**Figure S5.** Quantitative analysis of the relative fluorescence intensity of (A) IL-1β, (B) IL-6, (C) p16, and (D) p21. Data are expressed as mean ± SD (n = 3). Statistical significance was determined using one-way ANOVA followed by Tukey's post hoc test (*p < 0.05, **p < 0.01, ***p < 0.001).

**Figure S6.** Quantitative analysis of the relative fluorescence intensity of (A) COL II, (B) ACAN, (C) MMP 9, and (D) MMP 13. Data are expressed as mean ± SD (n = 3). Statistical significance was determined using one-way ANOVA followed by Tukey's post hoc test (*p < 0.05, **p < 0.01, ***p < 0.001).

**Figure S7.** Gene set enrichment analysis (GSEA) of (A) TNF signaling pathway, (B) NF-κB signaling pathway, (C) cellular senescence, and (D) collagen-containing extracellular matrix in the H₂O₂ versus Control comparison.

**Figure S8.** ^1^H-NMR spectra and proton signal assignments of OCS and HA-ADH-PBA. (A) ^1^H-NMR spectrum of OCS. (B) ^1^H-NMR spectrum of HA-ADH-PBA. The numbered peaks in each spectrum correspond to the specific proton signals indicated in the respective chemical structures.

**Figure S9.** Dynamic rheological frequency sweeps of the hydrogel at fixed strains of (A) 1% and (B) 10%.

**Figure S10.** Quantification of fluorescence indicators across different treatment groups. (A) JC-1 red/green fluorescence ratio (Aggregate/Monomer). (B) C11-BODIPY fluorescence ratio (Oxidized/Reduced). (C) Relative intensity of DCFH-DA fluorescence. Data are expressed as mean ± SD (n = 3). Statistical significance was determined using one-way ANOVA followed by Tukey's post hoc test (*p < 0.05, **p < 0.01, ***p < 0.001).

**Figure S11.** Quantitative analysis of the relative fluorescence intensity of (A) IL-1β, (B) IL-6, (C) p16, and (D) p21 across different treatment groups. Data are expressed as mean ± SD (n = 3). Statistical significance was determined using one-way ANOVA followed by Tukey's post hoc test (***P < 0.001).

**Figure S12.** Quantitative analysis of (A) the percentage of SA-β-gal positive cells, and the relative fluorescence intensity of (B) COL II and (C) MMP 13 across different treatment groups. Data are expressed as mean ± SD (n = 3). Statistical significance was determined using one-way ANOVA followed by Tukey's post hoc test (***P < 0.001).

**Figure S13.** Histological toxicity assessment of major organs. H&E staining of the heart, liver, spleen, lung, and kidney from rats treated with different formulations. Scale bar =20 µm.

**Figure S14.** Routine blood hematological analysis for in vivo biosafety assessment across different treatment groups. Quantitative analysis of (A) white blood cells, (B) neutrophils, (C) lymphocytes, (D) red blood cells, (E) hemoglobin, and (F) platelets from rats in the Control, IVDD, CDNVs, HAPOCS, and HAPOCS@CDNVs groups. The gray dashed lines denote the upper and lower limits of the normal physiological reference ranges for healthy rats. Data are expressed as mean ± SD (n = 3).

**Figure S15.** Serum biochemical analysis for in vivo biosafety evaluation across different treatment groups. Quantitative measurement of liver and kidney function indicators: (A) alanine aminotransferase (ALT), (B) aspartate aminotransferase (AST), (C) alkaline phosphatase (ALP), (D) albumin (ALB), (E) total protein (TP), (F) creatinine (CREA), (G) blood urea nitrogen (BUN), and (H) uric acid (UA) from rats in the Control, IVDD, CDNVs, HAPOCS, and HAPOCS@CDNVs groups. The gray dashed lines denote the upper and lower limits of the normal physiological reference ranges for healthy rats. Data are expressed as mean ± SD (n = 3).

**Table S1.** Primer sequences used for quantitative real-time PCR (*Homo sapiens*).

| **Gene Symbol** | **Gene Description** | **Forward Primer (5'–3')** | **Reverse Primer (5'–3')** |
| --- | --- | --- | --- |
| GAPDH | Glyceraldehyde-3-phosphate dehydrogenase | ACCCAGAAGACTGTGGATGG | TTCAGCTCAGGGATGACCTT |
| TNF-α | Tumor necrosis factor alpha | CCTCTCTCTAATCAGCCCTCTG | GAGGACCTGGGAGTAGATGAG |
| IL-1B | Interleukin 1 beta | ATGATGGCTTATTACAGTGGCAA | GTCGGAGATTCGTAGCTGGA |
| IL-6 | Interleukin 6 | ACTCACCTCTTCAGAACGAATTG | CCATCTTTGGAAGGTTCAGGTTG |
| CDKN2A (p16) | Cyclin-dependent kinase inhibitor 2A | GATCCAGGTGGGTAGAAGGTC | CCCCTGCAAACTTCGTCCT |
| CDKN1A (p21) | Cyclin-dependent kinase inhibitor 1A | TGTCCGTCAGAACCCATGC | AAAGTCGAAGTTCCATCGCTC |
| COL2A1 (COL II) | Collagen type II alpha 1 chain | TGGACGATCAGGCGAAACC | GCTGCGGATGCTCTCAATCT |
| MMP13 | Matrix metallopeptidase 13 | ACTGAGAGGCTCCGAGAAATG | GAACCCCGCATCTTGGCTT |
| ACAN | Aggrecan | ACTTCCGCTGGTCAGATGGA | TCTCGTGCCAGATCATCACC |
